# Polymer Encapsulated Framework Materials for Enhanced Gas Storage and Separations

**DOI:** 10.1021/acsmaterialsau.4c00109

**Published:** 2024-12-02

**Authors:** Grace E. B. Redwine, Wade A. Braunecker, Thomas Gennett

**Affiliations:** † Department of Chemistry, Colorado School of Mines, 1012 14th Street, Golden, Colorado 80401, United States; ‡ Chemistry and Nanoscience Center, National Renewable Energy Laboratory, 15013 Denver West Pkwy, Golden, Colorado 80401, United States

**Keywords:** covalent organic frameworks, metal organic frameworks, polymer encapsulated materials, polymer-coated materials, core−shell polymer composites, gas storage, gas separation, energy storage

## Abstract

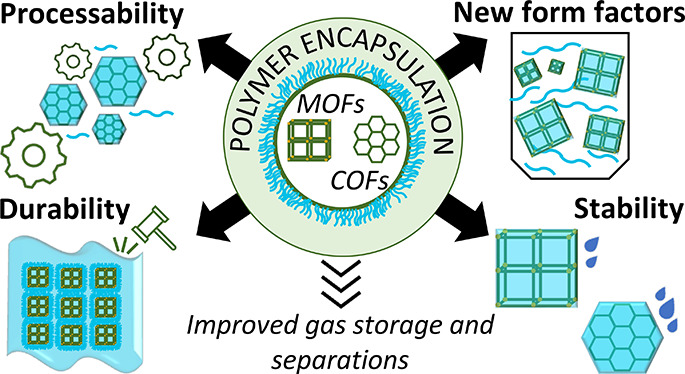

Within the broader field of energy storage, polymer-encapsulated framework (PEF) materials have witnessed remarkable growth in recent years, with transformative implications for diverse applications. This comprehensive review discusses in detail the latest advancements in the design, synthesis, and applications of PEFs in gas storage and separations. Following a thorough survey of existing literature, the article delves into mechanistic considerations and foundational principles governing PEF synthesis. Emphasis is placed on covalent and coordinative covalent grafting methods, physical blending, nonsolvent utilization, and various vapor deposition techniques. The discussion critically evaluates the advantages and disadvantages of these synthesis approaches, considering factors such as grafting density, coating thickness, and other physical properties relevant to processability and stability in comparison to traditional framework materials. Special attention is given to the impact of polymer coatings on gas adsorption analysis. Finally, notable accomplishments and advancements in the PEF field, including mixed matrix membrane (MMM) technology, improvements in framework form factors, and enhanced chemical and mechanical stability are summarized. This review concludes by offering valuable perspective for researchers, highlighting gaps and challenges that confront the current state-of-the-art in PEF materials, paving the way for future innovations that are poised to help address global energy challenges.

## 1 Introduction

In the past two decades, metal–organic frameworks (MOFs) and covalent–organic frameworks (COFs) have garnered attention for several exceptional attributes, including their high surface areas, tunable physicochemical properties, and versatile structures. These characteristics have driven progress in numerous fields, spanning energy storage, chemical separation technologies, drug delivery, and catalysis.^1−9^ Notably, framework materials have made a substantial impact on gas storage and separation. In recent sorbent and membrane literature, the key features of MOFs have enabled them to establish new gas storage capacity and separation efficiency records.^10−12^ It is evident that these highly functional materials have transformative potential for a wide diversity of applications. One critical prerequisite for broader implementation of framework materials on an industrial scale is enhancement of their durability and processability. This is especially important for those frameworks that are inherently fragile microcrystalline powders susceptible to degradation under environmental and mechanical forces.^13,14^ Due to this, some framework materials that exhibit promising behaviors in a controlled laboratory setting may not be suitable for industrial use.^13−15^ The advancement of polymer-framework composites, particularly polymer-encapsulated frameworks (PEFs), has resulted in significant breakthroughs in addressing these limitations, demonstrating numerous physicochemical improvements over pristine MOFs/COFs.

### 1.1 Background on Organic Framework Materials

MOFs and COFs are materials with porous framework structures formed from repeating structural units/motifs. MOFs are highly crystalline materials synthesized via the formation of coordinate covalent bonds between metal ions/clusters and organic ligands. COFs are ordered materials composed exclusively of light elements such as boron, carbon, nitrogen, and oxygen. In contrast to MOFs, the crystallinity of COFs is dependent on the dynamic covalent chemistry of the chosen ligands.^6−8^ The variety of metals and ligands from which to choose allows for exceptional tunability in MOFs and COFs. Properties such as pore size, reactivity, and stability can be modified and controlled by altering the metal–ligand (MOF) or ligand–ligand (COF) pairs. Many frameworks exhibit high adsorption capacity and/or selectivity for gases such as CO_2_ or H_2._^10−12,16−19^ Despite the progress made in recent years toward the synthesis of stable MOFs,^20^ they are historically prone to chemical instability due to the labile nature of metal–ligand coordination bonds.^15,21^ Many MOFs are specifically susceptible to hydrolysis, undergoing degradation in humid and aqueous environments.^22,23^ This decreases their effectiveness as gas separation materials due to the prevalence of humidity in gas streams and the ubiquity of water in the environment.^24^ MOF microcrystalline powders also tend to be mechanically fragile, which leads to brittleness, structural damage from handling, and hazardous dust formation.^13^ COFs are generally more stable than MOFs in harsh solvents and conditions, but still face challenges with processability.^25^ The combination of instability and poor processability makes it difficult to transition MOFs and COFs to practical engineering solutions for gas storage and separations, with only a limited number of MOFs being employed in large-scale industrial production and commercialization.^26−30^

### 1.2 Polymer-Framework Composites

**Figure 1:**
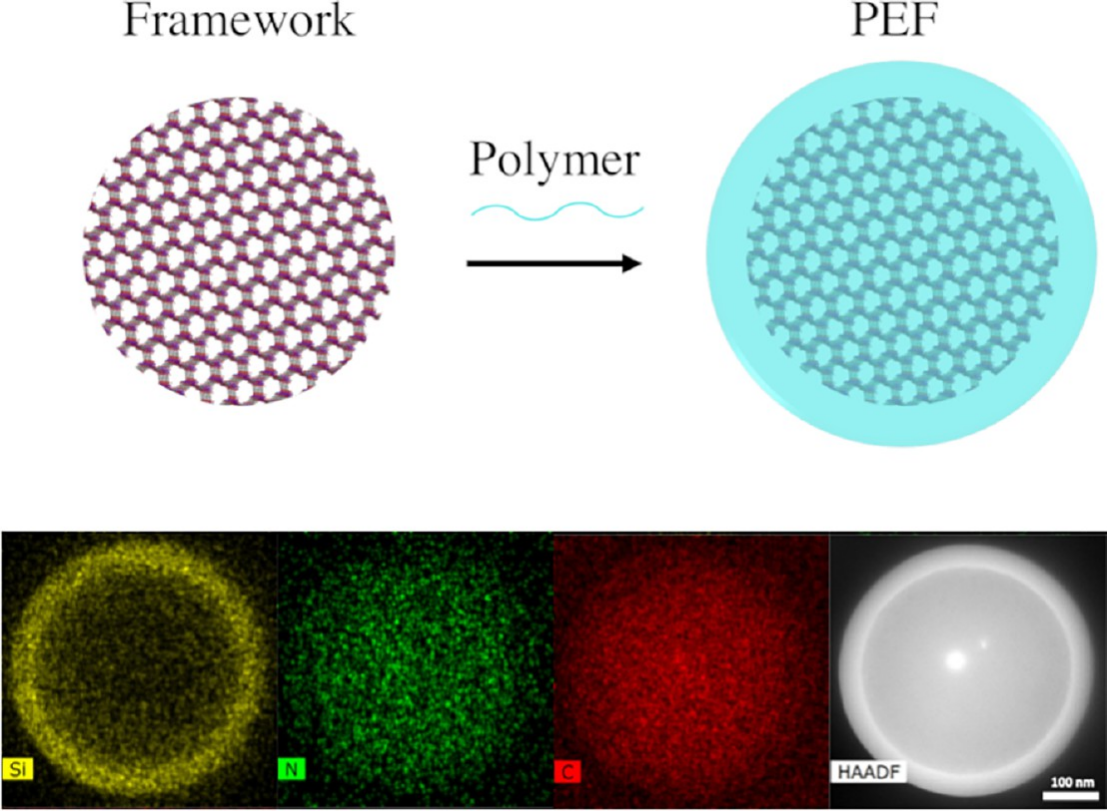
Scanning transmission electron microscopy (STEM) and STEM-energy dispersive X-ray spectroscopy (EDS) images of a polymer encapsulated framework (PEF) consisting of colloidal COF-301 particles with a poly(dimethylsiloxane) coating.^41^ Scale bar = 100 nm. The higher concentration of Si at the outer boundary of the spheroid, compared to the uniform distribution of C throughout both the coating and framework, suggests that the coating remains on the surface and does not penetrate or fill the framework pores. Adapted with permission under a Creative Commons CC-BY 4.0 from ref (41). Copyright 2024 American Chemical Society.

Developing new composite materials from MOFs/COFs and polymers is a relatively recent research area focused on addressing these practical challenges. Polymer-framework composites combine the traits of MOFs and COFs with the flexibility of polymers, resulting in improved processability and durability compared to the original framework materials. Several methods can be used to generate these hybrid materials, resulting in a range of chemical compositions, structural arrangements, and material characteristics. These methods range from simple blends with limited interaction between the framework and polymer, like mixed-matrix membranes (MMMs), to more complex approaches such as polymer grafting within the framework pores.^11,12,31−40^ Polymer-encapsulated frameworks (PEFs) are a specific type of composite that is achieved by encapsulating the framework particles with a polymer coating or shell (Figure 1). A distinct polymer shell and MOF/COF core differentiate these materials from polyMOFs and MOF-encapsulated polymers.

PEFs offer distinct advantages over other polymer-framework composites, including better compatibility between framework and polymer phases as well as maintaining the framework’s functional properties. In composites with minimal bonding between phases such as MMMs, issues may arise due to incompatibility between the crystalline (in)organic framework and soft, flexible polymer chains. Phase separation can result, straining the polymer chains and leading to defects such as voids, pore blockage, and material stiffening. These are all detrimental to the composite’s mechanical properties. The polymer shell bound to the surface of the nanoparticles in PEFs results in compatible polymer–polymer interactions between particles, minimizing defect formation. Concerning functionality, when a polymer is grafted from within or penetrates the framework’s pores, it can reduce the porosity of the material. However, by confining the polymer to the particle’s surface and forming a coating, PEFs preserve the interior nanoparticle porosity. As a result, PEFs emerge as a beneficial type of polymer-framework composite. This comprehensive exploration delves into PEF synthesis and properties documented in the literature. This work also presents an overview of some of the challenges associated with characterization of these materials, as well as advances in the field that have the potential to transform gas storage and separation technologies.

### 1.3 Overview of the Existing Review Literature

The field of polymer-framework composite materials has experienced significant growth, leading to a marked increase in review papers over the past 5–7 years. However, no comprehensive review exists specifically focused on core–shell polymer-framework composites for gas storage and separation applications. Various reviews cover polymer-framework hybrids, such as polyMOFs, framework-encapsulated polymers, mixed-matrix membranes (MMMs), MOF-organic hybrids, and inorganic-polymer hybrids, touching on PEFs but only briefly due to broader scopes.^31,42−50^ Other reviews focus on specific applications like bioimaging or drug delivery.^51−53^ Narrower reviews on MMMs, covalently bound MOF-polymer composites, and MOF-biopolymer composites mention PEFs incidentally but do not address the challenges specific to their synthesis and characterization.^34,37,54−62^ Concurrently, reviews on polymer-grafted nanoparticles are relevant to PEF formation but do not cover the unique challenges of preserving pore accessibility in MOFs or COFs during coating processes.^63−70^

This review focuses on synthetic strategies to tailor PEFs for improved gas storage and diffusion, offering a comprehensive analysis of their synthesis, characterization, and potential in gas separation, while also identifying underexplored areas in the field.

## 2 Polymer Coatings on Framework Materials—Mechanistic Considerations

**Scheme 1:**
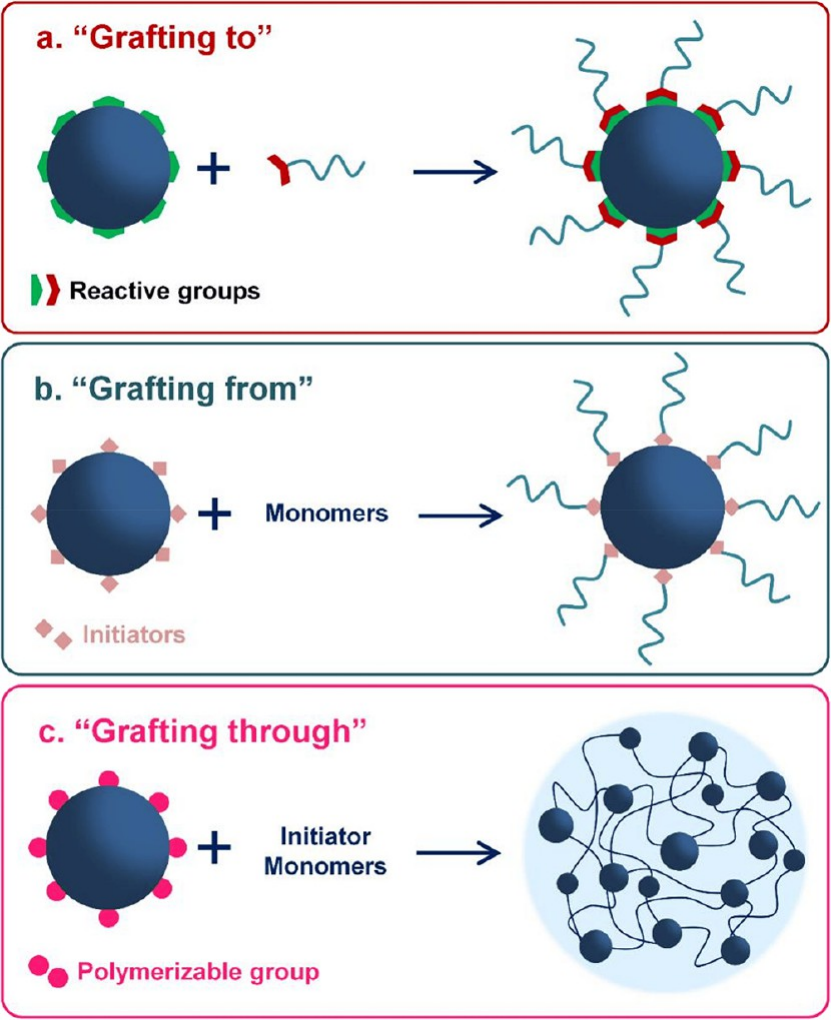
Strategies for Polymer Grafting Techniques Commonly Employed to Synthesize PEFs: (a) *Grafting to*, (b) *Grafting from*, and (c) *Grafting through* Reprinted with permission under a Creative Commons CC-BY 4.0 license from ref (72). Copyright 2018 MDPI.

For applications in gas storage and separations, key characteristics of framework materials are their high surface areas, large pore volumes, and available binding sites. While a tethered polymer coating can enhance material properties, infiltration of the polymer into the pore structure decreases surface area and can block adsorption sites. If the infiltration is substantial, the material is no longer useful for gas storage and separation. Therefore, the mechanism of polymer attachment is critical. Attachment mechanisms in the literature typically fall into one of three categories: *grafting from*, *grafting to*, or *grafting through* (Scheme 1).^71−73^ When the framework particle is functionalized in a way that allows it to act as a macroinitiator for polymer growth, the polymer coating is said to be applied using a *grafting from* mechanism.^41,74−81^ In cases where the framework acts as a macro-capping agent for a presynthesized polymer, the mechanism is considered *grafting to*.^82−89^ If the framework is functionalized to serve as a macromonomer or macro-cross-linking agent during polymerization, the coating process may be classified as *grafting through*.^90−96^

While these classifications serve as useful starting points, it is important to note that in some cases the mechanisms can be more nuanced and may not neatly fit into any single category, or they may span multiple categories. Even so, considering the advantages and disadvantages of each grafting technique can be helpful when designing a synthetic process for a material with a specific application in mind. For instance, in cases where a presynthesized polymer is affixed to the framework surface via chemi- or physisorption using a *grafting to* method, often excellent size-exclusion of the polymer from the framework’s interior is achieved. This is because the hydrodynamic volume of a random coiled polymer is typically substantially larger than the pores of the framework. However, due to steric repulsion between polymer chains, the overall grafting density on the particle’s surface after this technique tends to be rather low, which can lead to a more permeable coating. Conversely, in *grafting from* techniques, the grafting density is significantly higher, and lower permeability coatings are easier to attain. Depending on the pore size of the framework, *grafting from* can result in partial or complete pore filling as it is more challenging to size-exclude monomers than polymers. *Grafting through* techniques are subject to the same problems as *grafting from* but can produce particularly robust macro-gels for membrane applications, though it would be a poor choice for synthesizing discrete PEFs for fluid applications.

The bonds formed in these grafting techniques can be classified as coordinative covalent, covalent, or hydrogen bonding. Additionally, van der Waals interactions are occasionally employed in *grafting to* techniques. Each of these bonding mechanisms present their own challenges and advantages, over and above those of the specific grafting technique. In the following sections, we endeavor to unravel these intricacies, offering the reader a clearer perspective on the synthetic mechanisms available and their implications on corresponding material properties when contemplating a specific synthetic strategy for a new PEF material.

### 2.1 Coordinative Covalent Attachment

**Figure 2:**
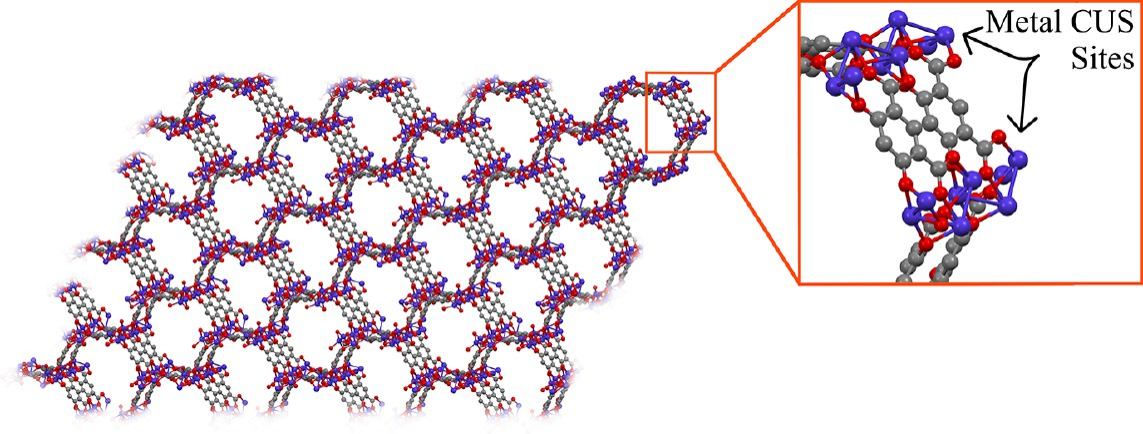
Coordinatively unsaturated metal sites (metal CUSs) occurring on the surface of a MOF nanoparticle, Co_2_(m-dobdc) [cobalt (blue), oxygen (red), carbon (gray)].

Coordinative covalent attachment in PEFs leverages the presence of incomplete ligand–metal coordination sites at the framework particle’s surface. In MOFs where metal ions/clusters form coordinative covalent bonds to ligands, coordinatively unsaturated sites (CUSs) can form in imperfections in the lattice or on the surface of the particle, at the termination of the framework structure, as shown in Figure 2. Metal CUSs are accessible to postsynthetic coordinative bond formation,^97,98^ and surface-specific postsynthetic modification can be implemented with strategic chelators that form bonds specific to these sites.^99−101^ Here, we discuss several strategies in the context of *grafting from* and *grafting to* techniques that take advantage of metal CUSs in MOFs.

These techniques are advantageous because the CUSs accessible to the polymer or initiator are almost entirely on the surface, with any lattice imperfections near the surface of the particle present in low concentrations. As a result, this group of PEFs retains most of their original porosity. *Grafting from* metal CUSs is a precise method to control location, thickness, and maximize grafting density of a coating. Even with a *grafting to* approach, use of metal CUS attachment results in much higher grafting density than a typical *grafting to* method. Through different polymers and molecular weights (*M*_w_) of the polymer coating—either the *M*_w_ of the polymer attached or the ratio of monomer in the grafting from method—these PEFs have versatile properties while retaining their original porosity.

#### Grafting from

Katayama et al. used a coordinative postsynthetic exchange strategy whereby they tethered initiator molecules to the surface of ZIF-8 in an example of a *grafting from* mechanism.^74^ They employed a robust living radical polymerization technique known as atom transfer radical polymerization (ATRP). In this work, an ATRP initiator molecule, designed for use with methacrylate-based polymers, was first synthesized from α-bromoisobutyryl bromide (BiBB) and histamine (his) dichloride to form his-BiBB. This initiator molecule contained an imidazolium group, which was then postsynthetically exchanged with imidazolium ligands on the surface of ZIF-8. This exchange and the subsequent polymerization were limited to the surface via size-exclusion, as ZIF-8 possesses a small pore aperture, between 3 and 4 Å.^102^ These exceptionally small pores enabled successful *grafting from* without any drawbacks associated with pore filling. The Brunauer–Emmett–Teller (BET) surface area decreased from 1625 m^2^/g to 1398 m^2^/g, which at 87 wt % loading corresponds to the nonporous mass. With the initiator tethered to the surface of the particles, the authors were able to form a well-defined coating via surface initiated (SI)-ATRP. The materials were then employed in the formation of robust, flexible monolayer films. The core–shell structure was critical to achieving continuous monolayers in these films. While this particular initiator is only applicable to frameworks with imidazolium ligands, this study provides a blueprint for using the postsynthetic exchange strategy to grow robust polymer coatings, assuming an initiator with size-exclusion properties that can exchange with the designated linkers of a MOF can be engineered.

Yang et al. also modified BiBB in order to graft a poly(methyl methacrylate) (PMMA) coating from a MOF (PCN-222).^103^ They proposed that functionalizing BiBB with an aminobenzoic acid moiety would allow its coordinative attachment to the metal CUSs of the MOF. This was followed by SI-ATRP of methyl methacrylate to form a PMMA coating. The resulting polymer composites’ mechanical properties were enhanced by adding PEFs, more so than pristine MOFs. N_2_ adsorption isotherms indicated that surface area was maintained following initiator attachment; however, it significantly decreased from approximately 1600 to just 300 m^2^/g after polymerization. Interestingly, the pore size distribution within the MOF was largely unchanged by the coating. The authors concluded that N_2_ adsorption into the MOF was partially blocked by the PMMA coating. Indeed, PMMA is glassy at cryogenic temperatures where N_2_ adsorption is typically measured. A partial or complete drop in N_2_ adsorption in these materials can be indicative of a dense and impermeable coating, *vide infra*. Although N_2_ adsorption at 77K can be hindered by some coatings that are impermeable at that temperature, it is still the most commonly used figure of merit for PEFs. When N_2_ adsorption is not blocked, the BET surface area before and after coating provides a reliable indicator of a surface-excluded coating. Other gas adsorption isotherms can be utilized at higher temperatures when N_2_ adsorption is blocked at cryogenic temperatures (i.e., CO_2_ at 273 K).

**Figure 3:**
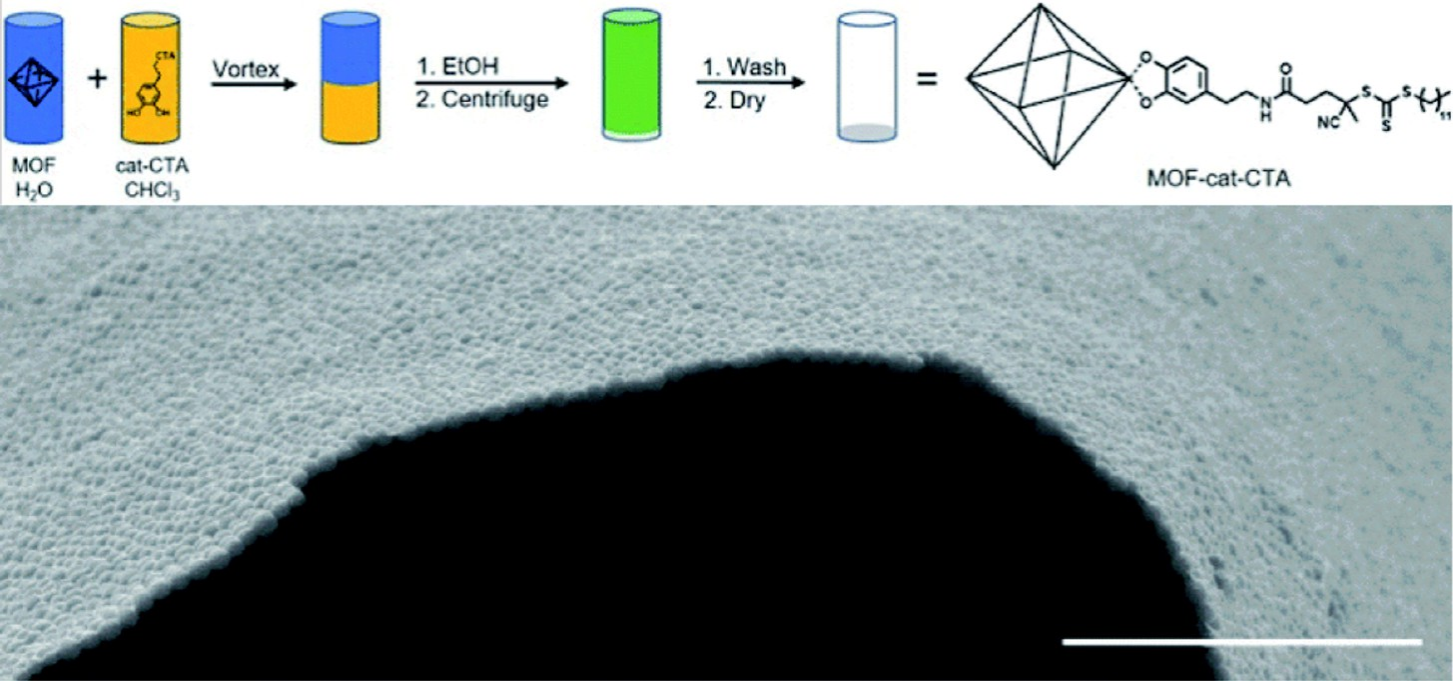
(Top) Reaction scheme of catechol-functionalized chain-transfer agent coordinatively binding metal CUS sites on MOF. (Bottom) Scanning Electron Microscopy (SEM) image of continuous monolayer formed from the polymer-coated UiO-66(Zr)-PMMA. Scale bar = 5 mm. Adapted with permission from ref (75). Copyright 2020 Royal Society of Chemistry.

Barcus and Cohen employed a catechol-based chain transfer agent (cat-CTA) with three different postsynthetically modified MOFs.^75^ The catechol chelated with partially unsaturated metal CUSs at the surface of the MOF (Figure 3). A surface-initiated living radical polymerization technique known as reversible addition–fragmentation chain-transfer (RAFT) was then employed to polymerize methyl methacrylate from the tethered CTA. This process led to the formation of robust coatings that facilitated self-assembly, resulting in single-layer, self-standing films with a high MOF loading. N_2_ adsorption and BET analysis revealed the coated particles retained most of the pristine MOFs surface area, decreasing from 1040 m^2^/g to 1015 m^2^/g for UiO-66(Zr)-PMMA and 692 m^2^/g for UiO-66-NH_2_–PMMA. Using a catechol as a ligand to coordinate with surface-exposed metal CUSs is a broadly applicable and very surface-specific method for anchoring initiator molecules to MOFs of various metals.

#### Grafting to

Rowe et al. also utilized partially exposed metal sites at the surface of their Gd MOFs as a coordinative attachment point in their PEFs, but they presynthesized their polymers using RAFT and employed the thiol end groups from the RAFT chain transfer agent for tethering the copolymer.^104^ The *grafting to* strategy resulted in consistent coatings of approximately 9 nm, improving the MOF stability in water and saline while enabling the materials to effectively target specific cells in a complex biological environment. They extended this approach to various other RAFT polymers and were able to control the thickness of the coating by controlling the molecular weight of the polymer.^105,106^ These RAFT copolymers were designed and synthesized to include fluorescent tags, reactive sites for therapeutic agents for treatment of disease, targeting ligands for biomolecular recognition, and a reactive thiol end group that could be tethered to the MOF contrast agent. While these PEFs were not employed for gas storage applications but used instead as MRI contrast agents in biological systems, we include this example here because it highlights the incredibly complex chemistry that is enabled by the RAFT polymerization technique.

Zimpel et al. also employed coordinative covalent bond formation via postsynthetic exchange in their work published in 2019.^107^ They coated zirconium fumarate MOF nanoparticles by mixing them in solution with various polymers, including poly(acrylic acid) , polyamidoamine, and branched polyethylenimine, in another example of a *grafting to* mechanism. The polymers containing carboxylic acid or amine groups were believed to replace the formic acid solvent molecules that were coordinatively bound to surface metal sites. This mechanism was partially confirmed by various spectroscopic methods monitoring the release of formic acid from the MOFs during polymer coating, while N_2_ adsorption isotherms indicated that the MOF’s surface area was at least somewhat preserved, decreasing from 736 m^2^/g to between 543 m^2^/g to 400 m^2^/g depending on the polymer.

### 2.2 Covalent Attachment

**Scheme 2:**
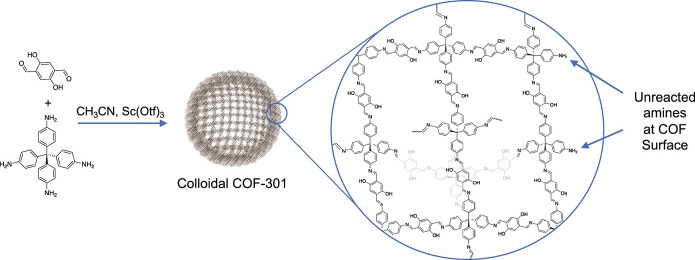
Synthesis of Colloidal COF-301 via Imine Linkage, Resulting in Unreacted Ligand (Amine) Functional Groups at the Surface of the Particle Adapted with permission under a Creative Commons CC-BY 4.0 from ref (41). Copyright 2024 American Chemical Society.

While coordinative covalent bond attachment is an effective strategy for synthesizing PEFs, it is inherently limited to MOFs, as the core structure of COFs are exclusively made of light elements and lack metal sites. Covalent attachment strategies could be considered more versatile as they can be employed to modify both COFs and MOFs. The multidentate ligands in a framework structure such as a MOF or COF will have available binding sites at the surface which can act as accessible chelators or functional groups, akin to metal CUSs (Scheme 2). Indeed, surface-limited postsynthetic modification on framework particles has been conducted by forming covalent bonds with surface ligands.^108,109^ Functional groups distributed throughout the framework but not directly engaged in the primary bonding making up the framework structure, could also be utilized to covalently attach a coating. However, if not correctly engineered with a size-exclusion component, the latter approach can result in a pore-filled material.

Covalent attachments are very robust, enable access to a broad range of chemistries, and can be applied to a wider variety of framework materials than coordinative covalent attachment strategies. Ligand CUSs, similar to metal CUSs, are highly effective as attachment sites for forming porous PEFs due to their surface specificity. When attaching covalently to non-CUS sites, increasing the size of the polymer or initiator helps reduce pore-filling, allowing covalent attachment to be as effective as coordinative attachment.

Covalent *grafting-from* is a promising method for creating PEFs, but its success hinges on effective surface-exclusion techniques. If a ligand CUS or macromonomer are utilized, the coatings formed are robust and surface-excluded, with controllable grafting density and thickness. This technique has also enabled innovative polymerization methods, such as light-activation of benzene groups in MOF linkers to form free radicals, and anionic polymerization from crystal edges initiated by electron-donating guests within the framework pores. Covalent *grafting to* is a simpler approach for forming surface-excluded PEFs, as polymer molecules are larger than initiators, making the process more straightforward. They are typically more facile and less expensive than *grafting from*. The main drawback to *grafting to* methods is the much lower grafting densities, but if a highly dense coating is not required this is not a significant issue. By adding reactive sites to the surface of the framework prior to *grafting to*, the grafting density can also be increased. *Grafting through* techniques are ideal for forming materials intended for membranes, as they excel at attaching and dispersing nanoparticles in membranes or fibers. While grafting-through requires additional processing when used for coated nanoparticles in solid form, with appropriate surface-exclusion strategies, it is one of the best methods for creating well-dispersed and mechanically superior mixed-matrix membranes with high loadings.

Covalent attachment methods range widely in chemical bonds formed and frameworks used, and in effectiveness. Here, we will examine the literature with a focus on the unique approaches and on successful porous PEF formation.

#### Grafting from

Mow et al. covalently tethered a macromolecular initiator consisting of a copolymer with ATRP initiator (bromoisobutyryloxy) groups and anchoring (benzaldehyde) groups to the surface of COF-301 via condensation (imine linking) with available surface amine groups on COF-301 (Scheme 2).^41^ This was followed by SI-ATRP of a poly(dimethylsiloxane) (PDMS)-methacrylate terminated monomer, forming a dense bottlebrush coating around the colloidal COFs. This surface-specific strategy combined with a macromonomer size-excluded from the COF’s small pores (∼6 Å) resulted in well-retained porosity. This was observed via CO_2_ adsorption isotherms, as the coating was glassy and impermeable to N_2_ at cryogenic temperatures. The CO_2_ adsorption decreased by approximately 20% in the coated material, most of which can be attributed to the polymer mass. Intriguingly, the dense coating also afforded a novel method of H_2_ storage, in which the H_2_ is kinetically trapped at cryogenic temperatures from diffusing through the coating. The resulting PEFs were able to form permanently porous liquids in poly(ethylene glycol) (PEG-200) and in PDMS, while the PEG infiltrated the uncoated COFs over a matter of days, reducing the porosity.

**Figure 4:**
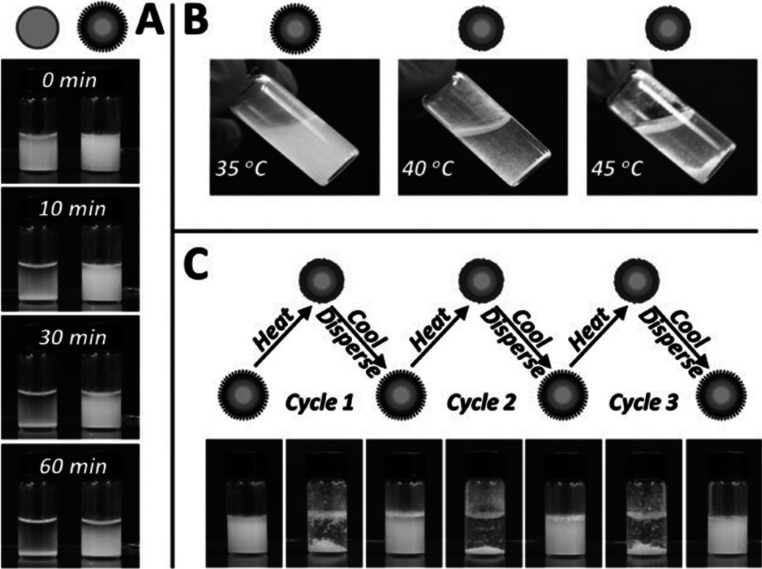
(a) Comparison of the dispersibility of MOF (left) versus PEF (right) in water at room temperature. (b) Temperature dependence of PEF dispersibility in water. (c) The reversible dispersibility of the PEF in water after heating and cooling cycles. Reprinted with permission from ref (76). Copyright 2014 Wiley-VCH Verlag GmbH & Co. KGaA, Weinheim.

Liu et al. covalently attached the initiator BiBB to MIL-101(Al)-NH_2_ to the primary amine pendant groups distributed throughout the framework structure.^76^ The pore sizes of this MOF are quite large at 25 and 30 Å, making it likely that the initiator was integrated throughout the framework.^110^ While the surface area of the material was not analyzed, this system was being studied for its dispersion behavior above and below lower critical solution temperature. Indeed, after polymerization of a random copolymer of 2-(2-methoxyethoxy)ethyl methacrylate and oligo(ethylene glycol) methacrylate, the surface and solution properties of the MOF drastically changed and were termed “reversibly dispersible” in water with respect to temperature (Figure 4).

Xie et al. covalently attached the common ATRP initiator BiBB to the -NH_2_ moieties on UiO-66-NH_2_ via the reaction of the acid bromide groups on BiBB with the primary amines decorating the framework.^77^ It should be noted that BiBB is a small molecule, so it is unlikely that it was completely retained on the surface of the particles. However, ATRP was conducted with a macromonomer, poly(ethylene glycol) methyl ether methacrylate with a molecular weight (M_W_) of 475 g/mol. From the perspective of the PEG macromonomer, the polymerization occurred via a *grafting through* technique, while also being *grafted from* the surface of the MOF. Due to this, the growing brush polymer was likely size-excluded because of UiO-66-NH_2_’s small pores. As this work did not include surface area analysis, it is difficult to determine whether this method formed a surface-constrained polymer coating.

Tabatabaii et al. grafted lauryl methacrylate brushes from UiO-66-NH_2_ by covalently reacting a monochlorosilane-terminated initiator (11-(chlorodimethylsilyl)undecenyl bromoisobutyrate) with the amine groups on the MOF.^78^ A reduction in surface area was observed at this stage, dropping from approximately 900 to 550 m^2^/g. SI-ATRP of poly(lauryl methacrylate) was then conducted to form a coating, after which the surface area further dropped to 200 m^2^/g. The resulting material improved water-based separation processes through the hybridization of a porous, rigid MOF with hydrophobic, flexible polymer coatings.

Wang et al. covalently tethered a chain-transfer agent (CTA) to ZIF-8-NH_2_ through the amine functionalities of the porous material in order to implement SI-RAFT of poly(*N*-(2-((2,2,2-trifluoroethyl)sulfinyl)ethyl)acrylamide) (PFSAM).^111^ The CTA was proposed to be size-excluded from the 6.2 Å pores. N_2_ adsorption isotherms and BET analysis indicated a moderate loss of surface area (1949 m^2^/g to 1336 m^2^/g) that could partially be attributed to addition of nonporous mass. The subsequent coating was then employed to control the diffusion of small molecules in a set of drug delivery experiments, highlighting the power of PEFs for potential separation applications.

**Scheme 3:**
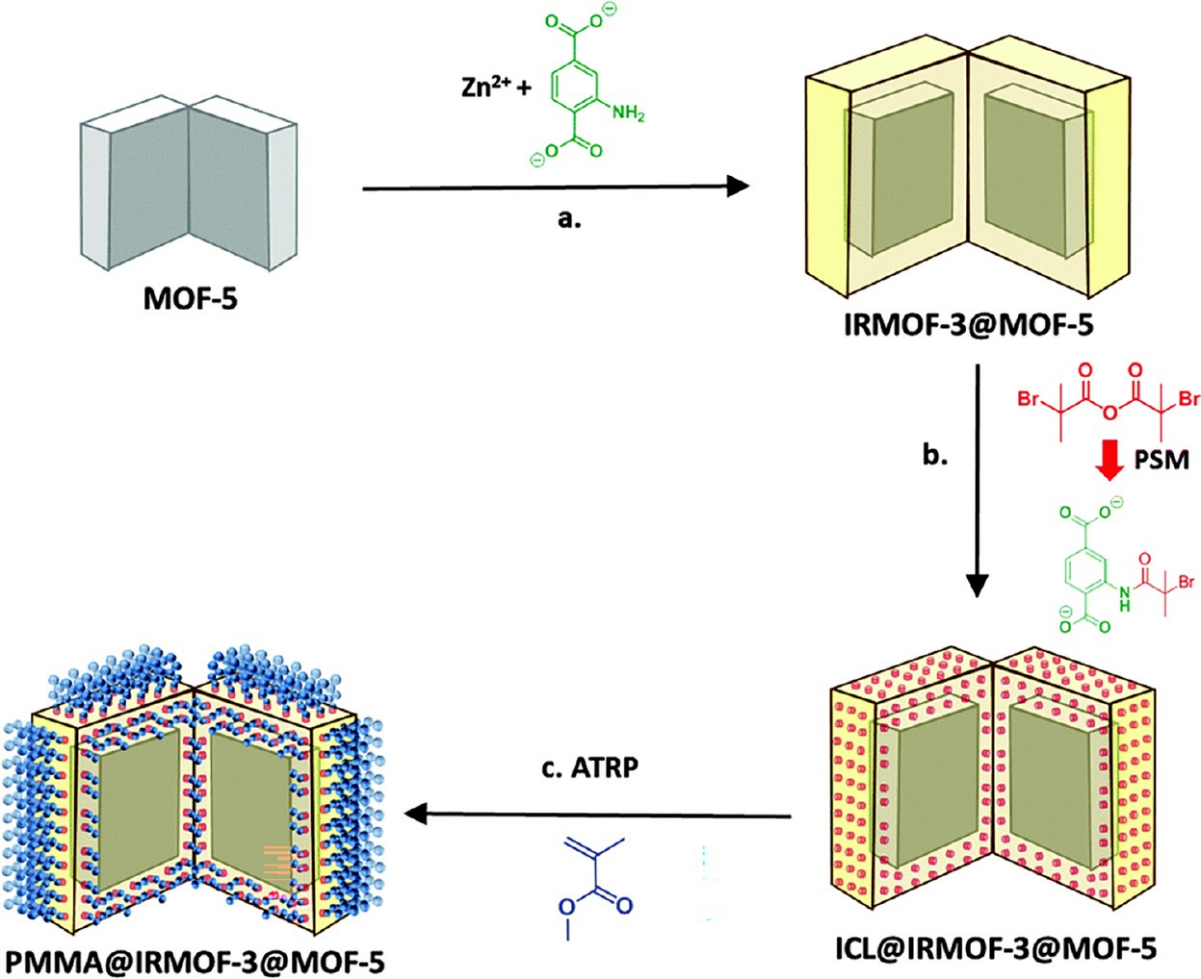
Synthetic Route to PMMA@IRMOF-3@MOF-5 Showing Both the Core and Shell Chemistry Reprinted with permission from ref (79). Copyright 2015 Royal Society of Chemistry.

McDonald et al. used a unique approach to maintaining an open pore PEF while employing a *grafting from* technique. A *grafting from* approach was specifically desired to increase grafting density, but a high surface area and open pore volume of a large pore MOF was also desired. Thus, they first created a core–shell MOF@MOF structure, growing an IRMOF-3 shell around a core of MOF-5 (Scheme 3).^79^ The initiator (2-bromoisobutyric anhydride) established covalent bonds with the amine groups in the shell MOF. ATRP was then used to grow a polymer@MOF@MOF structure with the polymerization of MMA throughout the outer MOF. Notably, the strategy was successful in retaining the polymer primarily on the surface of the outer MOF as well, as N_2_ adsorption and BET analysis showed a total decrease from 3530 m^2^/g to 2289 m^2^/g, which is less than would be expected if the entire outer MOF was pore-filled with polymer.

Hou et al. grafted a PMMA coating from several MOFs (UiO-66, UiO-66-NH_2_, MIL-125(Ti), MOF-5, IRMOF-3, and ZIF-8) by placing each in a solution of bulk monomer (MMA) and using UV light to induce free radical polymerization from the MOF linkers by breaking H–C bonds in the benzene rings.^80^ SEM images showed thickly coated nanoparticles, and the MOFs’ surface area decreased by approximately one-third. This matched the additional nonporous weight of the polymer as indicated by thermogravimetric analysis (TGA), signifying a core–shell structure although the polymerization strategy was not surface-specific.

Similarly, Takashima et al. installed a reducing agent, *N,N*-dimethylaniline, inside the pores of a napthalenediimide MOF, ([Zn_2_(bdc)_2_(dpNDI)]_*n*_). As an electron donating guest, the *N,N*-dimethylaniline molecule could induce electron transfer to the MOF linkers and generate radical anions at the crystal surfaces that in turn could initiate polymerization in the presence of vinyl monomers.^81^ Successful coatings of styrene, methyl methacrylate, and divinylbenzene were applied. CO_2_ adsorption isotherms showed negligible change after coating, indicating that the porosity of the material was preserved after coating, and further investigation showed that the PEF material was resistant to hydrolysis when submerged in aqueous mixtures.

#### Grafting to

Arguably the most common technique employed to date in the synthesis of PEFs has been *grafting to* with the formation of tethered covalent bonds. Size exclusion of large polymers from many framework pores makes this technique quite versatile, and covalent bonds tend to result in robust PEF materials.

**Figure 5:**
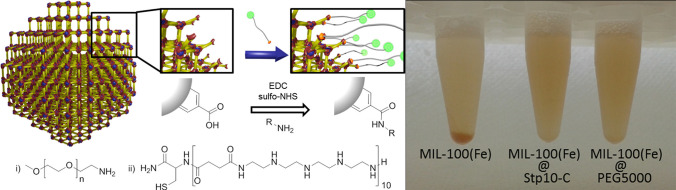
Illustration of polymer coating mechanism via peptide coupling to MIL-100(Fe) (left) and images showing the colloidal stability of coated MOF particles (right). Adapted with permission from ref (82). Copyright 2016 American Chemical Society.

Zimpel et al. grafted selected aminopolymers to a MOF via a peptide coupling reaction to surface carboxyl ligands on MIL-100(Fe) in 2016 (Figure 5).^82^ The resulting particles became stable as colloids and were hydrophobic, qualitatively consistent with being coated. The authors also confirmed the existence of the bond formation between polymer and framework via nuclear magnetic resonance (NMR). A modest decrease in surface area from 1905 m^2^/g to 1338 m^2^/g and 1432 m^2^/g for the two polymers was partially attributed to pore blocking by the polymer.

**Figure 6:**
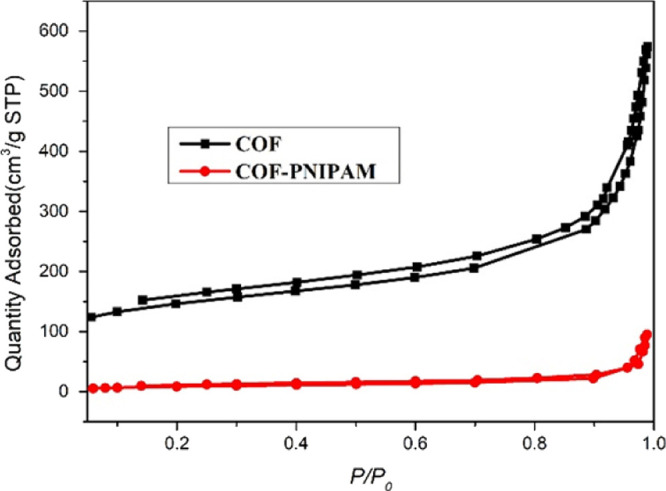
N_2_ adsorption analysis showing a reduction in amount of N_2_ adsorbed correlating to a change in surface area from ∼506 to ∼34 m^2^/g after coating. Adapted with permission from ref (83). Copyright 2020 The Chemical Society Located in Taipei & Wiley-VCH Verlag GmbH & Co. KGaA, Weinheim.

Zhang et al. grafted poly(*N*-isopropylacrylamide) prepared by RAFT with terminal carboxyl groups to a melamine Schiff-base COF with surface amine ligands.^83^ This strategy was well conceived for synthesizing a core–shell structure that maintains its porosity, as it utilized both size-exclusion *via grafting to* and a covalent, surface-exclusive bonding scheme. A drastic change in surface area was observed after coating (from ∼506 to ∼34 m^2^/g) (Figure 6). The resulting surface area was too low to completely attribute to the addition of nonporous polymer mass. However, the authors only use N_2_ isotherms to characterize the porosity and surface area of the material, and it is not possible to tell from those measurements alone whether the polymer may have infiltrated the pores of the material, or if a dense coating that is glassy at cryogenic temperatures is precluding N_2_ from adsorbing.

**Scheme 4:**
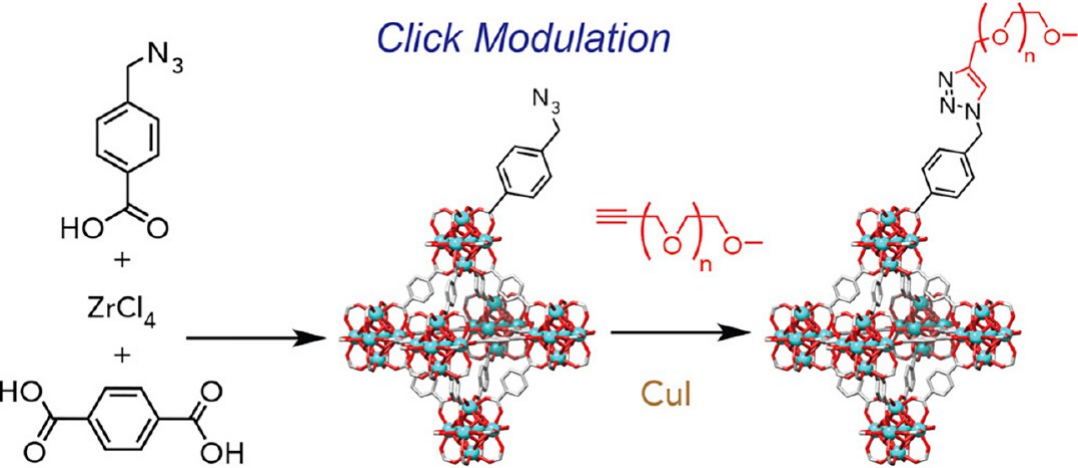
Click Modulation Chemistry to Form PEG-Coated UiO-66 Reprinted with permission under a Creative Commons CC-BY-NC-ND license.^89^. Copyright 2017 Elsevier.

Abánades Lázaro et al. used click chemistry to install a polymer coating on UiO-66.^89^ This method involved preparing azide moieties for postsynthetic modification that endowed the MOF surface with click chemistry capabilities. After installing the azide functionalities via postsynthetic modification, alkyne-functionalized PEG macromonomers were grafted to the MOF via copper-catalyzed azide–alkyne cycloaddition (CuAAC) (Scheme 4).^112^ Despite lacking a specific surface-excluding strategy in the postsynthetic modification step, surface area analysis confirmed the coating was mostly retained at the surface. The BET surface area decreased from 1565 m^2^/g to 865 m^2^/g and 521 m^2^/g for the different PEG polymers, with 22–23 wt % attributed to the polymers via TGA.

A significant subset of polymer coatings in the literature, including numerous examples employing *grafting to* strategies, involve the MOF UiO-66-NH_2_. The ligand in this MOF is 2-aminoterephthalic acid, where the coordinative bonds occur between the zirconium metal and carboxylic acid functional groups. This leaves primary amine groups dispersed throughout the nanoparticle structure. The pore size of UiO-66-NH_2_ is about 6 Å, small enough that many polymers and even macromonomers are easily size-excluded.^113,114^ For example, Nagata et al. attached an *N*-hydroxysuccinimide functionalized poly(*N*-isopropylacrylamide) (PNIPAM-NHS) polymer to UiO-66-NH_2_ through the reaction of the primary amines and the NHS ester moiety.^115^ Due to the size of the PNIPAM-NHS (2000 Mw), the polymer is constrained to the surface of the MOF. This was confirmed with experiments verifying the retention of reactive sites inside the pores. The coated MOF was found to be thermoresponsive due to the properties of PNIPAM, introducing the idea of functional, “smart” PEFs that could be applicable in energy storage.

Yao et al. reacted UiO-66-NH_2_ with isocyanate-terminated polyurethane to form a membrane.^116^ The reaction of the isocyanate end groups with the amine sites on the MOFs created a covalently bound MOF-urea membrane. The presynthesized polymer was in principle size-excluded from the pores of the material. However, CO_2_ adsorption isotherms of the resulting membranes indicate some pore-filling may have occurred, as the decrease in CO_2_ uptake is larger than can be attributed to the MOF wt % in the membrane. The membrane formed was durable and flexible due to the high interfacial interactions.

Katayama et al. published a mechanism involving coordinative postsynthetic exchange in UiO-66-NH_2_ followed by a covalent *grafting to* polymer coating.^84^ 2-allylterephthalic acid (H_2_bdc-allyl) was employed, leaving allyl moieties on the surface of the MOF. Polydimethylsiloxane (PDMS) was then grafted to the allyl groups via hydrosilylation. Surface area analysis showed little to no change after coating (from 783 m^2^/g to 734 m^2^/g), indicating a successful surface postsynthetic exchange (PSE) step. Significant gas separation improvement was observed in the MMMs formed from the PEFs, due to the greatly decreased interfacial defects in the membrane as opposed to one formed with the pristine MOFs.

Satheeshkumar et al. implemented a strategy to form a MMM of UiO-66 by first synthesizing a MOF from 2-vinyl-1,4-dicarboxylic acid linkers (UiO-66 CH=CH_2_).^85^ The authors then used a radical initiated thiol–ene click reaction to form a highly cross-linked membrane of divinyl functionalized poly(ethylene glycol), vinyl functionalized MOF, and thiol cross-linking agents. The crystalline structure of the MOF was maintained throughout the MMM formation. While surface area analysis was not performed on the MMM, gas permeability was significantly enhanced compared to a pristine polymeric membrane, attributed to porous space in the MOF.

Li et al. designed a method in which the MOF was grown in solution from metal ions and organic linkers, along with a presynthesized polymer that was end-functionalized with linker groups. The unique, one-pot method was dubbed polymer–metal–organic framework self-assembly (PMOFSA),^86^ and it bears some of the hallmarks of a *grafting to* mechanism. As MOF particles begin to form, the modified poly(ethylene glycol) chains coordinate with the metal ions at an early stage of growth in what becomes the surface of the particle. The formation of a polymer shell prevents aggregation and precipitation of the MOF particles as they grow larger and less water-soluble. Various characterization techniques were employed to corroborate the proposed mechanism. Small-angle neutron scattering (SANS) measurements supported a core-polymer shell structure, while dynamic light scattering (DLS) confirmed the small, monodisperse nature of particles when the polymer-linker was used. In contrast, a synthesis without the polymer-linker led to visibly aggregated particles.

**Figure 7:**
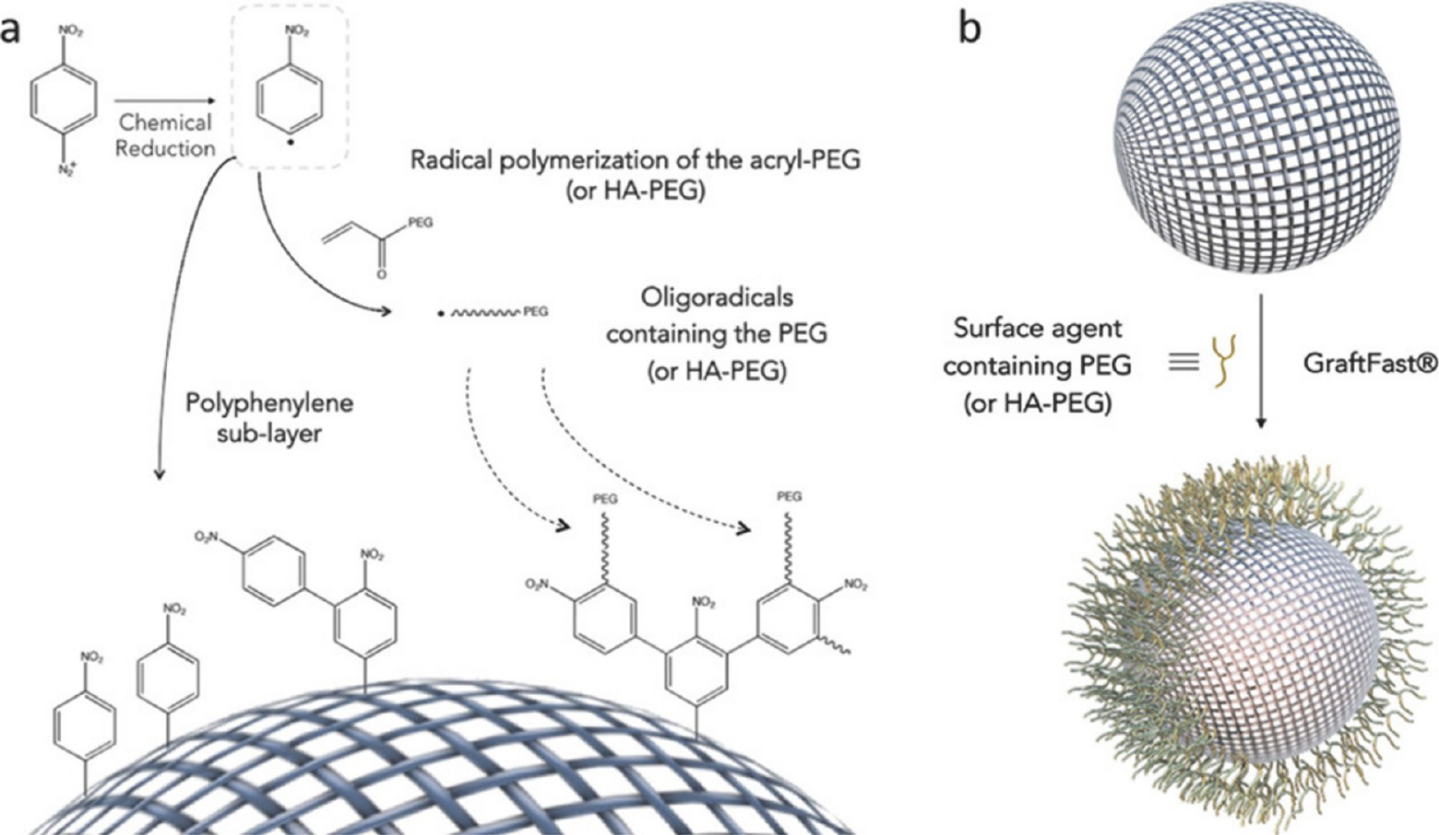
GraftFast technique for polymer coatings used by Giménez-Marqués et al. on MIL-100(Fe). (a) Scheme showing mechanism of reaction. (b) Graphical representation of coating formed through GraftFast. Reprinted with permission from ref (87). Copyright 2018 Wiley-VCH Verlag GmbH & Co. KGaA, Weinheim.

We also mention here the so-called GraftFast technique,^117^ a patented surface coating method employed recently by Giménez-Marqués et al. to functionalize the surface of MIL-100(Fe) (Figure 7).^87^ The proposed mechanism for this technique involves a number of steps, including 1) the generation of active aryl radical species from the reduction of nitrobenzene diazonium salts; 2) the chemisorption of some of those aryl radicals to a MOF surface to give a nitro-polyphenylene-like layer; 3) the polymerization of PEG macromonomers in solution, also initiated by aryl radical species; and 4) reaction of the radical PEG macromonomer with nitro-polyphenylene layer on the MOF to graft the PEG to the surface of the MOF. Surface area analysis showed the effectiveness of this method at forming a robust polymer coating as the BET surface areas decreased only from 1590 m^2^/g to 1470 m^2^/g, and the coated particles showed enhanced colloidal stability in water. Benzaqui et al. also used GraftFast to coat ZIF-8 nanoparticles with acryl-PEG (480 and 5000 g/mol).^88^ The coated nanoparticles were then placed in a matrix as a porous filler to create highly compatible MMMs. The coated particles also showed improved colloidal stability with surface area decreasing from 1700 m^2^/g to 1200 m^2^/g, indicating primarily surface-excluded polymer

#### Grafting through

**Figure 8:**
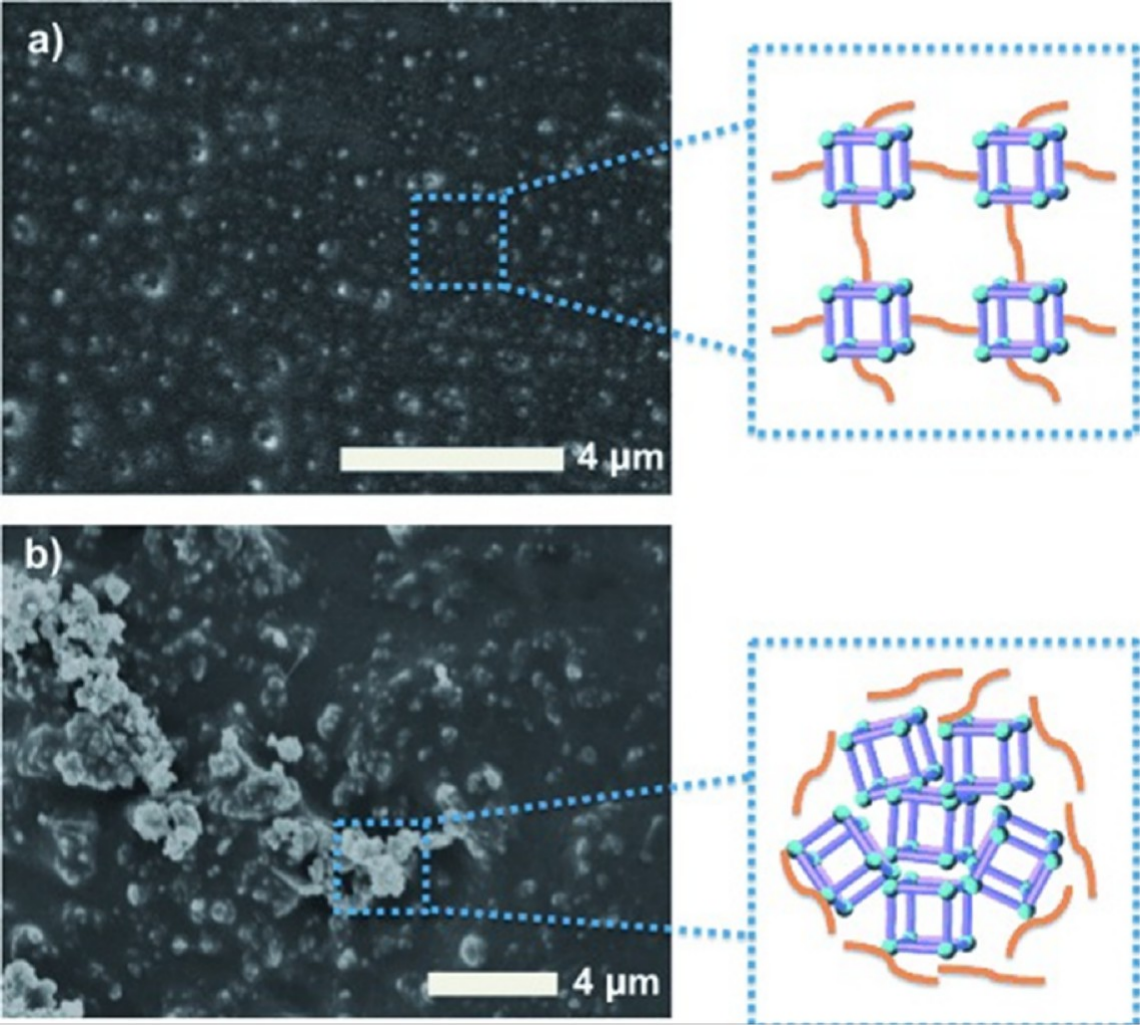
Difference in interfacial defects observed with (a) PEFs and (b) neat MOFs as porous fillers in an MMM. Reprinted with permission from ref (90). Copyright 2015 Wiley-VCH Verlag GmbH & Co. KGaA, Weinheim.

Zhang et al. formed a polymer-coated membrane by functionalizing the surface of UiO-66-NH_2_ with methacrylic anhydride to create a MOF-macromonomer. This material could then be cophotopolymerized in solution using a *grafting through* technique with butyl methacrylate to essentially form MOF-polymer networks that functioned as highly robust membranes that would not phase separate (Figure 8 a.).^90^ The surface area analysis showed a slight decrease from 854 m^2^/g to 675 m^2^/g.These membranes were studied for the removal of heavy metal ions from water. Compared to a membrane composed of unfunctionalized MOF dispersed in poly(butyl methacrylate) that formed large nonselective voids (Figure 8 b.), the separation performance of the coated MOF membrane improved dramatically.

Jiang et al. attached a pH-responsive polymer coating with UiO-66-NH_2_ using a similar method of functionalization with methacrylic anhydride followed by the *grafting through* copolymerization of the MOF and 2-[(diethylamino)ethyl methacrylate].^91^ While crystallinity and morphology was shown to be retained after coating via powder X-ray diffraction (PXRD) and electron microscopy, surface area analysis was not performed. The covalently bound polymer coating enabled pH-dependent dispersion and emulsifying behavior, showcasing the broad range of possible PEF material properties.

Rabiee et al. performed a postsynthetic modification of UiO-66-NH_2_ with glycidyl methacrylate (GMA).^92^ Using either free radical polymerization or a RAFT agent to control polymerization in two *grafting through* techniques, poly(hydroxyethyl methacrylate) (p(HEMA)) and p(NIPAM) coated MOFs were then synthesized. While surface area analysis was not conducted, the resulting material exhibited enhanced performance as a nanocarrier for drug and gene delivery applications. The coating increased the stability and durability of the MOF nanocarriers, allowing for high drug loadings and reducing toxicity.

Molavi et al. also functionalized UiO-66-NH_2_ with GMA before grafting a poly(methyl methacrylate) coating from the surface.^93^ Surface area analysis via N_2_ adsorption indicated that the surface area nearly disappeared after polymerization (from 965 m^2^/g to 13 m^2^/g). This could indicate a similar phenomenon mentioned above, where cryogenic temperatures required for N_2_ adsorption result in glassy polymer coatings which impede adsorption. The PMMA coated MOFs resulted in improvements in gas selectivity and durability in MMMs through improved interfacial interaction and dispersion.

Gao et al. implemented a size-excluded postsynthetic modification strategy by functionalizing UiO-66-NH_2_ with norbornene.^94^ This functionalization was followed by a copolymerization of the monomer functionalized MOF with other norbornene monomers *via grafting through* with ring-opening metathesis polymerization (ROMP). No change in the surface area was observed following the postsynthetic modification, indicating the initial functionalization and subsequent polymerization were pore excluded. Like most materials formed with *grafting through* techniques, the coated MOF formed a highly dispersed, stable MMM. This work is one of the few that have also reported scale-up attempts. The authors report one of the largest single-component MMMs in the literature (98 × 165 cm) for factory-scale gas separations.

In an example of utilizing a step-growth process for growing a polymer coating, Wang et al. functionalized UiO-66-NH_2_ in such a way as to leave a tethered anhydride species available for further reaction.^118^ Subsequent step-growth polymerization with dianhydride and diamine monomers in the presence of the monoanhydride-functionalized MOF incorporated the MOF into a growing polyimide. We include this example alongside other *grafting through* polymerizations, as the MOF is functionalized with multiple anhydride moieties and can therefore effectively serve as a macro-cross-linking agent. However, step-growth processes arguably cannot be classified the same ways as chain-growth polymerizations and are subject to their own nuanced optimization procedures. Regardless, 5–10 nm thick polyimide coatings were efficiently grown on these MOFs, and the success of the coating was confirmed by surface area analysis (a slight decrease from 1068 m^2^/g to 907 m^2^/g after coating), electron microscopy, and PXRD. The material was employed in efficient MMMs.

**Figure 9:**
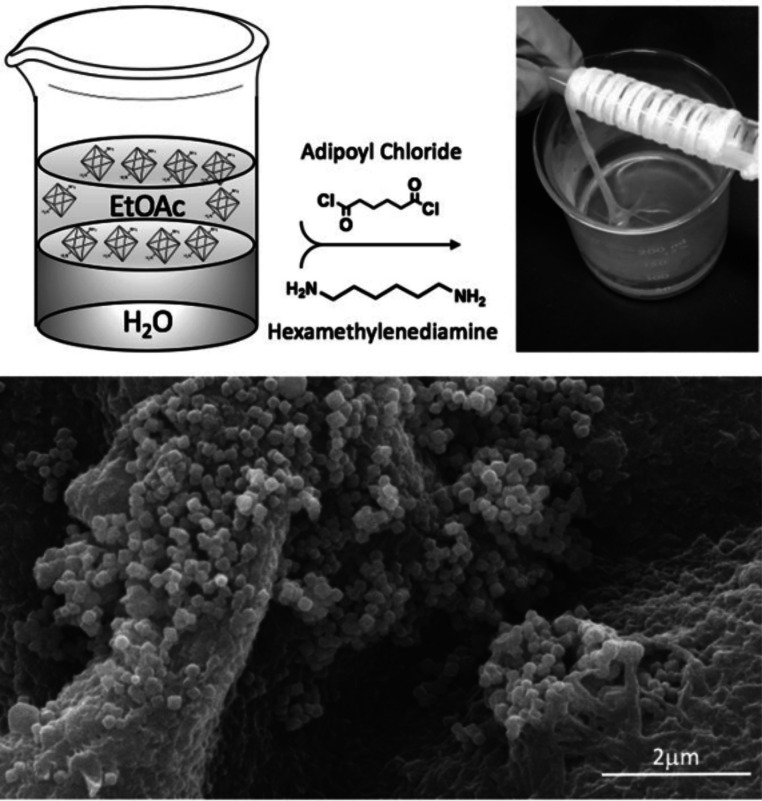
(Top) Solution interface fabrication of MOF-nylon fiber. (Bottom) SEM image of MOF-nylon fiber. Reprinted with permission from ref (96). Copyright 2019 Wiley-VCH Verlag GmbH & Co. KGaA, Weinheim.

Step-growth polymerization during PEF synthesis was also employed by Kalaj et al. to encapsulate UiO-66-NH_2_ within a nylon-6,6 fiber.^96^ Postsynthetic modification of the MOF with adipoyl chloride was employed to tether acid chloride reactive sites to the particles. This material was then copolymerized with hexamethylenediamine and additional adipoyl chloride via a solution interface method to form a polyamide (nylon-6,6) fiber full of covalently tethered MOF (Figure 9). Although surface area analysis using N_2_ adsorption isotherms revealed a decrease in pore accessibility of 63% compared to free MOF particles, it was still considerably higher than that of a noncovalent blend of MOF and polymer, which decreased by 84%. Notably, the resulting PEF material exhibited significantly greater catalytic activity for chemical and environmental remediation applications than the corresponding polymer/MOF blends.

### 2.3 Polymer Adsorption

Coordination and covalent attachment strategies, while effective, can be synthetically complex and reliant on unsaturated surface moieties for maintaining a surface-exclusive coating. A simpler approach to create PEFs is to use weaker intermolecular forces such as hydrogen bonding, van der Waals interactions, or electrostatic forces for attaching initiator molecules, monomers, or polymers to the framework. This can be achieved through a variety of methods that facilitate the adsorption of these components to the framework surface. These techniques are more straightforward than the coordinative or covalent methods discussed above. This approach is especially attractive for large-scale industrial applications due to its lower resource requirements and synthetic accessibility.

Adsorption-based techniques range from physical blending of components to polymerization of monomer in solution with the framework nanoparticle. The primary characteristic of these techniques are their low grafting density and their straightforward, rapid, and scalable procedures. For applications which require enhanced chemical stability, such as gas separation and storage of humid gas streams, adsorptive techniques have been very effective. Indeed, most of the works discussed in this section are aimed at improving the framework chemical stability. Mechanical durability can also be improved through these low-density coatings, specifically when forming pellets for large-scale applications. Creative and especially scalable, rapid coatings are found using this technique, including thermal deposition of a coating and nonsolvent induced rapid coating deposition. For PEF applications which do not require extensive control of the polymer dynamics of the coating, adsorptive coatings are the most facile strategy.

#### Physical Blending of Components

Qian et al. coated MOF nanoparticles by mixing a commercially available organosilicone dissolved in heptane with three different MOFs (NH_2_-MIL-125(Ti), ZIF-67, and HKUST-1).^119^ Following the workup process, transmission electron microscopy-energy dispersive X-ray spectroscopy (TEM-EDS) images distinctly revealed the retention of silicon on the particle surfaces. This coating rendered the nanoparticles hydrophobic, imparting resistance to degradation by water. Simultaneously, the MOFs retained their structural integrity, crystallinity, and surface area. In another similar example, Fernandez et al. adsorbed the commercially available polymer Pluronic P123 to the surface of two well-known MOFs (MIL-101(Cr) and Ni_2_(dobdc)), relying on attractive forces between the polymer’s polar moieties (ethers and alcohols) to adsorb the polymer to the surface of the MOFs.^120^ This coating also decreased the water uptake of the MOFs, resulting in increased stability and cyclability in humid gas streams. The high surface area and CO_2_ adsorption capabilities were also preserved relative to that of the pristine MOFs. This facile method of physically blending framework nanoparticles with polymers in solution has subsequently been utilized by several additional research groups in the literature to generate water-stable PEFs that maintain their porosity.^121,122^

**Scheme 5:**
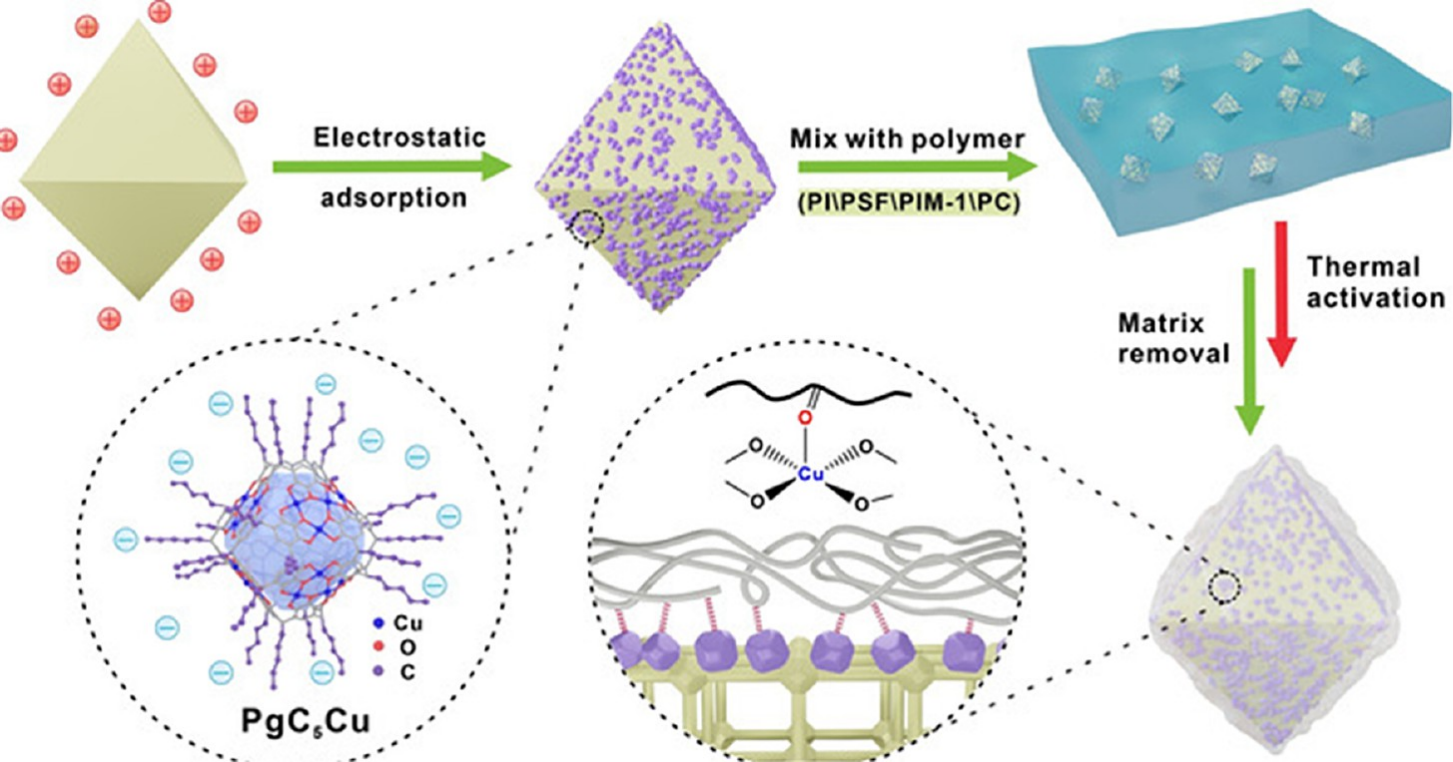
The MONC-Mediated Polymer Coating Method Reprinted with permission from ref (123). Copyright 2021 Wiley-VCH GmbH.

Li et al. also employed a physical adsorption mechanism, enhancing it with additional cross-linking to create a robust PEF.^123^ They first electrostatically adsorbed a negatively charged metal–organic nanocapsule (MONC) with open Cu metal sites to a positively charged MOF (Scheme 5), confirming successful adsorption with surface potential measurements. The functionalized MOF was subsequently mixed in solution with a polymer containing polar functional groups, such as polyimide, polysulfone, polycarbonate, etc. Following workup, the materials were heat treated to expel solvent molecules. At this stage, the polar functional groups in the polymer coating formed coordinative cross-links with the open Cu sites at the surface of the MONC-coated MOF, creating a uniform sub-10 nm coating. This technique resulted in near ideal dispersity of the coated MOF when suspended in a matrix of the same polymer, and the surface area was unchanged after coating. The technique is attractive because it provides a versatile means for creating a robust, coordinated cross-linked coating that is not MOF specific.

Kang et al. utilized a straightforward adsorption method involving PDMS coatings.^124^ While initially demonstrated on a diamine-grafted MOF for carbon capture applications, this technique is applicable to a broad range of framework-based materials. The process was demonstrated on Mn_2_(dobpdc) grafted with N-ethylethylenediamine, where PDMS was added dropwise to the bulk MOF powder. The mixture was flame-sealed in a glass tube and annealed at 235 °C for 6 h. N_2_ isotherms showed no uptake, and CO_2_ adsorption isotherms indicated reduced adsorption postcoating by approximately 50%, implying potential polymer infiltration into pores. However, this method resulted in hydrophobic MOFs with enhanced resilience in humid CO_2_ streams. Although not explicitly discussed in this context, it is noteworthy that PDMS has been observed by other research groups to undergo thermal cross-linking under similar conditions, resulting in exceptionally durable PDMS coatings (*vide infra*).

Kim et al. employed a synergistic approach, combining hydrogen bonding and coordination bonding, to coat a zirconium MOF (PCN-224) with hyaluronic acid (HA) biopolymer.^125^ Computational results supported the hypothesis that HA coordinates with the MOF nanoparticles through a combination of these bonding mechanisms. Potential bonding interactions include those between the carboxylic acid moieties on HA and the hydroxyl groups on the MOF, as well as between the carboxylic acid moieties of HA and the coordinatively unsaturated Zr sites near the MOF surface. While the surface area was not analyzed postcoating, the resulting coating formed an enzyme-responsive barrier, showcasing its potential utility for controlled small molecule and/or drug release from the MOF.

**Figure 10:**
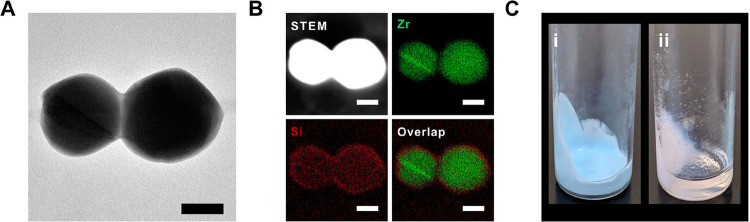
(a) TEM and (b) Scanning transmission electron microscopy (STEM-EDS) images of a polymer-coated MOF, showing the coating distinctly on the surface of each particle. (c) Porous liquids formed from the PEF (i) versus MOF (ii) showing permanent dispersibility of the PEF in a liquid polymer. Scale bars = 200 nm. Adapted with permission from ref (127). Copyright 2019 American Chemical Society.

He et al. published a method for coating a series of MOF colloids by mixing them with a presynthesized copolymer containing carboxylic acid groups and ATRP initiating groups (bromoisobutyrates).^126^ It was proposed that the copolymer adsorbed onto the surface of the colloidal MOFs via intramolecular hydrogen bonding. SI-ATRP could then be conducted in a *grafting from* reaction using styrene or (meth)acrylic monomers to grow a second, thicker polymer shell around the MOF. If a diacrylate cross-linking agent was employed during SI-ATRP, a robust cross-linked coating was formed. The technique enabled both high grafting density and controllable coating thicknesses. N_2_ isotherms showed blocked N_2_ uptake at cryogenic temperatures, while CO_2_ isotherms showed 80–90% retention of porosity depending on the polymer. Employing this technique, the research group subsequently synthesized and examined coated MOF materials as “porous liquids”.^127^ In this innovative approach, the PEF maintains permanent porosity even when suspended in liquid polymers like PDMS (Figure 10). This method has gained popularity for imparting MOFs with the unique property of fluidity, expanding the potential applications of these materials.

#### Polymerization in Solution

Several physical adsorption coating techniques have been implemented by conducting the synthesis of PEFs in solution. In most cases, this is accomplished through the polymerization of monomers in solution which then leads to adsorption of the growing polymer onto the framework surface. These synthetic approaches have resulted in facile formation of core–shell PEFs, even without a specific surface-exclusion strategy.

Xiao et al. combined a melamine Schiff-base COF with dopamine under alkaline conditions, results in the spontaneous self-polymerization of dopamine in solution.^128^ The polydopamine is observed to adsorb efficiently to the surface of the COF, likely through hydrogen bonding. N_2_ adsorption isotherms showed a decrease in adsorption capacity from 207 m^2^/g to 110 m^2^/g after the coating was applied, which may be due to the small dopamine monomer becoming trapped in the pores. The polydopamine coating conferred chelating sites to the COF, improving its ability to purify metal ions from wastewater. This is another example of a polymer coating imparting functionality, rather than just processability to a framework material—an aspect that could have impact in the realm of energy storage materials.

**Figure 11:**
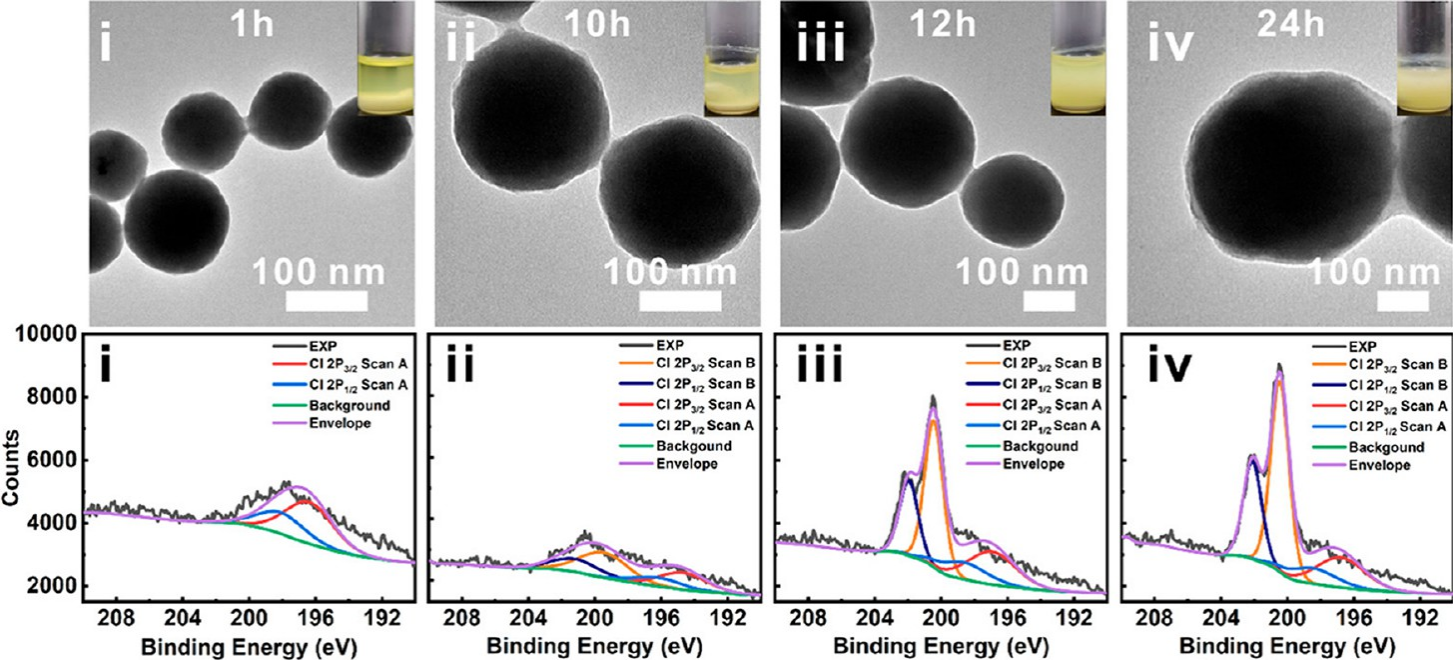
(Top) TEM images and (bottom) high-resolution XPS spectra of MOF-801@P-1 at different reaction times: (i) 1 h; (ii) 10 h; (iii) 12 h; (iv) 24 h. The insets show the colloidal stability of the MOF suspension after 3 h of settling time. Reprinted with permission from ref (129). Copyright 2021 American Chemical Society.

Li et al. recently introduced a strategy termed Polymerization-Induced Surface Adsorption (PISA), utilizing both adsorption and hydrogen bonding to attach growing polymers to MOF surfaces.^129^ The authors conducted a RAFT polymerization in the presence of MOF-801 nanoparticles and a hydrogen bond-donating monomer, 2-hydroxyethyl methacrylate, that could be copolymerized with a range of other monomers. The authors proposed that as the polymer chains grew in solution, MOF/polymer interfacial interactions gradually increased, resulting in eventual adsorption of the polymer to the MOF surface through hydrogen bonding and weak intermolecular forces. Aliquots were systematically taken during the 24 h polymerization, from which MOF particles were washed, isolated, and analyzed by X-ray photoelectron spectroscopy (XPS). In a copolymerization that employed 4-chlorostyrene and 2-hydroxyethyl methacrylate, XPS was used to monitor the appearance of a Cl peak associated with the growing polymer. This peak was first observed at the 12 h mark, which corresponded to an increase in MOF colloidal stability that also occurred at the 12 h mark (Figure 11). These results support the polymerization-induced adsorption hypothesis. While lacking a specific pore-exclusion strategy, this method effectively maintained the MOF surface area, as although N_2_ adsorption was blocked, CO_2_ adsorption isotherms were unchanged after coating.

Ding and Jiang published a variation on the PISA method above, aiming for thinner framework coatings.^130^ Using monomers with carbonyl moieties to enhance the hydrogen bond formation with the HKUST-1 surface, they conducted a free radical polymerization initiated by azobis(isobutyronitrile) (AIBN). The authors propose a similar mechanism to PISA, in which the growing polymer adsorbed onto the framework surface. The N_2_ adsorption isotherm of the coated material was unchanged from the pristine framework; however, the hydrolytic stability of the MOF was greatly improved. The resulting coatings were observed to be 15–25 nm thick depending on polymerization time, indicating the success of the initial effort of using a PISA mechanism to create coatings in a range of desired thickness.

#### Use of Nonsolvents

**Figure 12:**
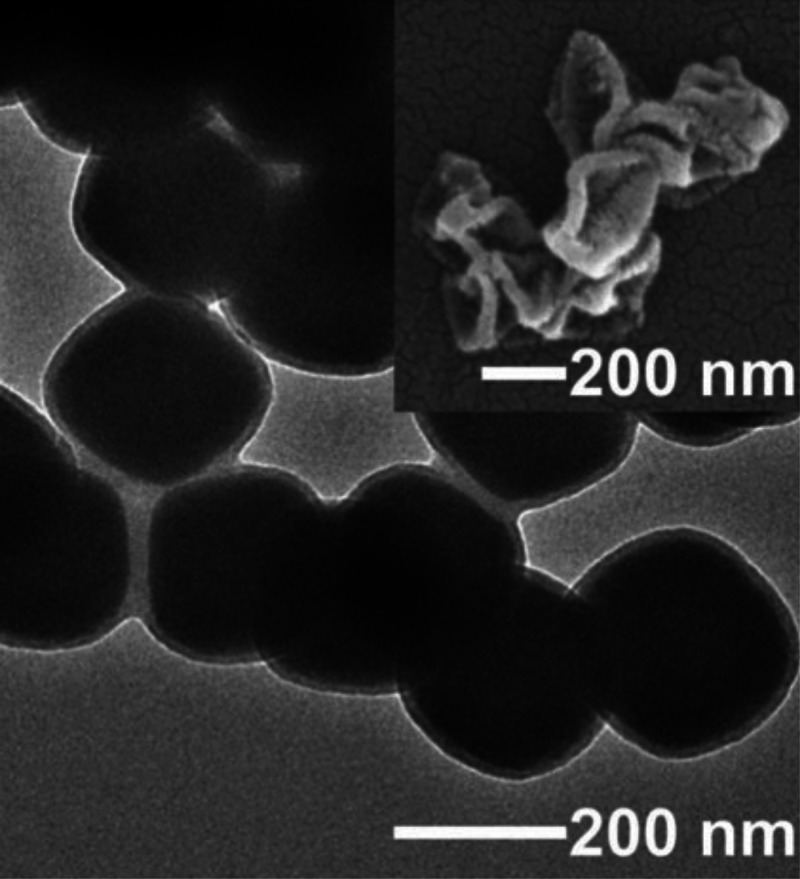
TEM image of polyimide-coated MIL-101(Cr) after NISAP. Inset: SEM image of polymer shell after MOF digestion. Reprinted with permission from ref (131). Copyright 2022 American Chemical Society.

A solution-based mechanism termed nonsolvent-induced surface-aimed polymerization (NISAP) was developed by Wang et al.^131^ The MOF MIL-101(Cr) and chosen monomers (multidentate amines and dianhydrides) were dissolved in a “good” solvent (DCM), followed by the addition of excess “bad” solvent (petroleum ether). The phase separation induced by the nonsolvent led to a rapid increase in monomer concentration at the surface of the MOF. Subsequent polycondensation resulted in a uniform, sub-10 nm thick, cross-linked polymeric coating within minutes (Figure 12). After correcting for the added mass of polymer, the N_2_ isotherms are unchanged after coating.

**Figure 13:**
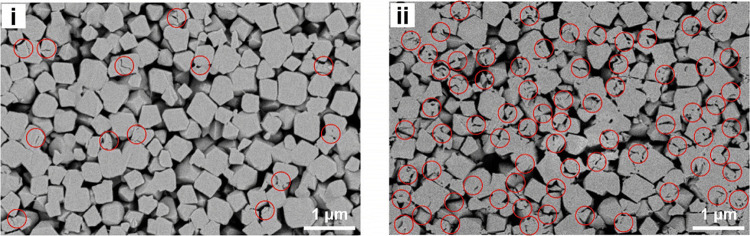
SEM images of cross-sectional slices of a pellet from coated MOF-801 (i) vs uncoated MOF-801 (ii), indicating stress points and mechanical damage in red circles. Reprinted with permission from ref (132). Copyright 2021 Royal Society of Chemistry.

A similar method termed nonsolvent induced surface deposition (NISD) was developed by the same group.^132^ However, instead of monomers, presynthesized polymers were adsorbed to the surface of the MOF nanoparticles through the addition of a nonsolvent to the polymer/MOF mixture. The ensuing coated MOFs could be pressed into pellets with much less damage to their structure when compared to the pristine MOF pellets (Figure 13). The pellets retained the surface area and functionality of the pristine MOF while showing great improvement in both chemical and mechanical stability.

#### Vapor Deposition Techniques

**Scheme 6:**
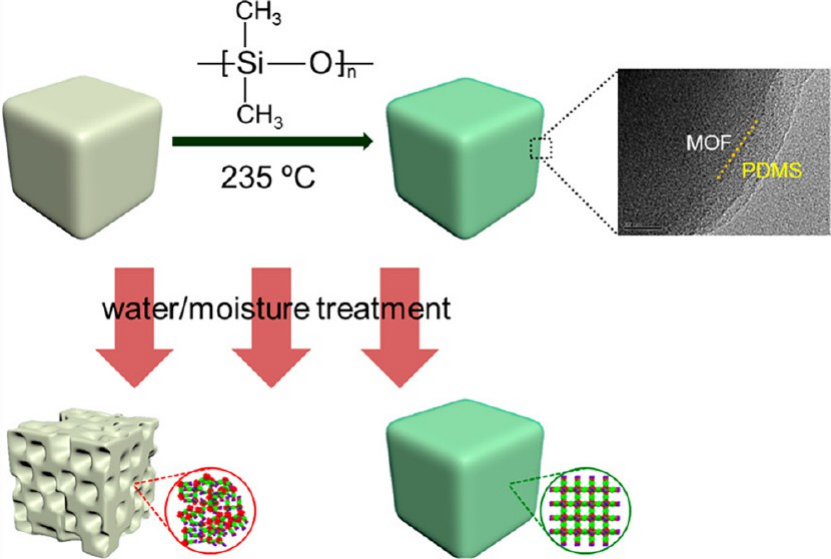
Vapor Deposition of PDMS onto MOFs to form PEFs Reprinted with permission from ref (136). Copyright American Chemical Society 2014.

Thermal vapor deposition (TVD) has emerged as a compelling strategy for coating framework materials, relying on weak intermolecular forces and demonstrating ease of application. In the studies discussed, polydimethylsiloxane (PDMS) serves as the source of the polymeric coating. When framework particles are heated slightly above ∼200 °C alongside liquid PDMS or a PDMS stamp, either oligomeric PDMS vapor or volatile silicone molecules, resulting from PDMS thermal breakdown, are believed to adsorb onto the framework surfaces (Scheme 6). After adsorption, the molecules are hypothesized to further cross-link, forming a robust PDMS coating. TVD, now widely used to coat various porous materials, stands out as a facile method for creating hydrophobic materials.^68,133−135^

Zhang et al. used the TVD technique to form a PDMS coating on the surface of MOF nanoparticles.^136^ They heated the MOFs (MOF-5, HKUST-1, and ZnBT) in the presence of a PDMS stamp. This resulted in the creation of MOF powders with 10 nm hydrophobic coatings that retained nearly 100% of their active surface area. Jin et al. formed Mg-MOF-74 coated with PDMS using this same technique, resulting in the material remaining stable in water for over one year.^137^ Huang et al. also formed hydrophobic UiO-66/Pd MOF composites using TVD.^138^ The application of the coating not only increased the recyclability of the catalyst by preventing the aggregation of nanoparticles but also elevated its performance in reactions with hydrophobic reactants. The hydrophobic nature of the surface promoted the concentration of hydrophobic substrates around the catalytic site. Furthermore, the coating contributed to enhanced selectivity by effectively sieving reactants with varying levels of wettability.

### 2.4 Discussion

Organizing coatings by the mechanism of attachment is useful for fundamentally understanding the interaction between coating and framework. However, these mechanisms comprise a wide range of methodologies. The coating method affects the physiochemical properties of the coating which in turn affects the properties of the overall material, whether through particle–particle interactions or through the membrane properties of the polymer shell.

#### Coating Thickness

**Figure 14:**
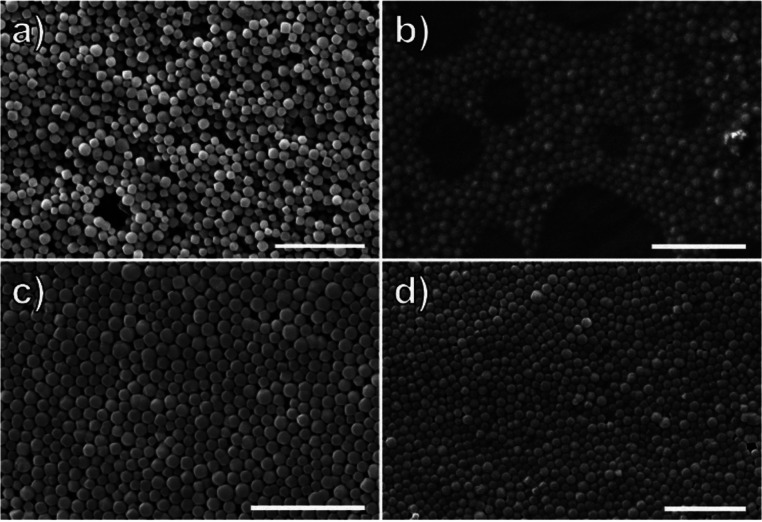
Improvement in packing and interfacial interactions with increasing polymer brush molecular weight in coated MOF particles. (a) *M*_n_ = 6300 g/mol. (b) *M*_n_ = 8300 g/mol. (c) *M*_n_ = 59 000 g/mol. (d) *M*_n_ = 138 000 g/mol. Scale bars = 3 μm. Reprinted with permission from ref (75). Copyright 2020 Royal Society of Chemistry.

Being able to control the thickness of the coating is important as the coating thickness can affect the properties of the resulting material. One group observed that they could not form self-supporting, single-layer membranes until their polymer coating exceeded 138 000 g/mol due to insufficient particle–particle interactions (Figure 14).^75^ Another observed that a 20 nm thick coating showed greater chemical stability when exposed to H_2_SO_4_ than an 8 nm thick coating.^131^ Others noticed dispersity and colloidal stability increased with increased coating thickness.^87,126,129^

Many methods of polymer coating theoretically allow some optimization of coating thickness via selection of polymer/framework material ratio, monomer/initiator ratio, starting polymer molecular weight, or polymerization time. In practice, only a handful of papers report being able to experimentally control coating thickness.^75,126,129−132,137,138^ Methods utilizing an initiator tethered to a metal CUS or surface ligand followed by surface-initiated (*grafting from)* polymerization exhibit the most control over coating thickness, showing an increase in thickness proportional to increasing monomer concentration or increasing polymerization time.^75,126^ Polymerization-induced surface adsorption (PISA) methods also resulted in some amount of control, allowing the formation of thin (∼15 nm) and thick (25 and 85 nm, respectively) coatings with increased monomer concentrations or polymerization time.^129,130^ Thermal vapor deposition (TVD) lent itself to controlling thickness with respect to deposition time.^137,138^ Nonsolvent-induced polymerization or deposition methods also resulted in some level of control over the coating thickness, resulting in thin and thick coatings by altering the concentration of monomers or polymer in the solution.^131,132^ Select *grafting to* methods which utilized highly surface-specific binding demonstrated some control over the thickness of the coating as well. In these cases, grafting higher molecular weight polymers to the framework resulted in thicker coatings.^87,105^ When considering all these results, broadly speaking, surface-initiated *grafting from* techniques offer the most precision control over coating thickness.

#### Grafting Density

**Scheme 7:**
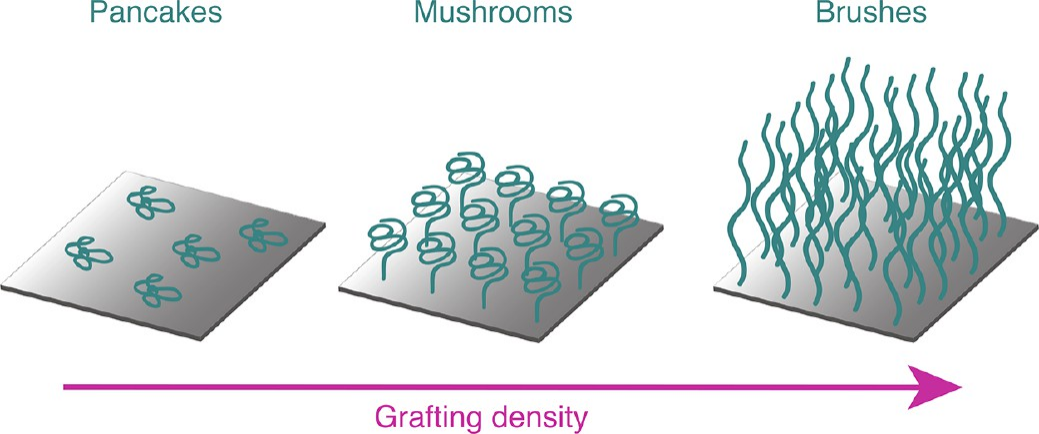
The Conformation of Grafted Polymer Chains with Increasing Grafting Density Flat “pancake” conformations without entanglement appear at very low grafting density, while “mushroom” conformations appear at moderate grafting density when the polymer chains can entangle, and at high grafting density brush-like conformations appear when the polymer chains are dense enough to be strained, decreasing entanglement. Reprinted with permission under a CC-BY 4.0 license from ref (150). Copyright 2020 MDPI.

The grafting density of a coating onto a nanoparticle significantly influences the material properties by altering polymer conformation as well as the coated particle’s ability to form entanglements with other particles or matrices, impacting both mechanical properties and gas diffusion (Scheme 7). High grafting densities (0.6–1.0 chains/nm^2^) stiffen the polymer corona and reduce entanglement with other chains, while moderate densities (0.3–0.5 chains/nm^2^) increase interpenetration and entanglement.^139−146^ In membranes, moderate grafting densities can produce flexible membranes while higher densities can lead to stiffer mechanical properties and increased gas permeability due to the free volume around the stiffened polymer coating.^139,147,148^ If the grafting density is quite low (0.05 chains/nm^2^), the particles may aggregate in solution, behaving like unmodified nanoparticles.^144,149^

**Figure 15:**
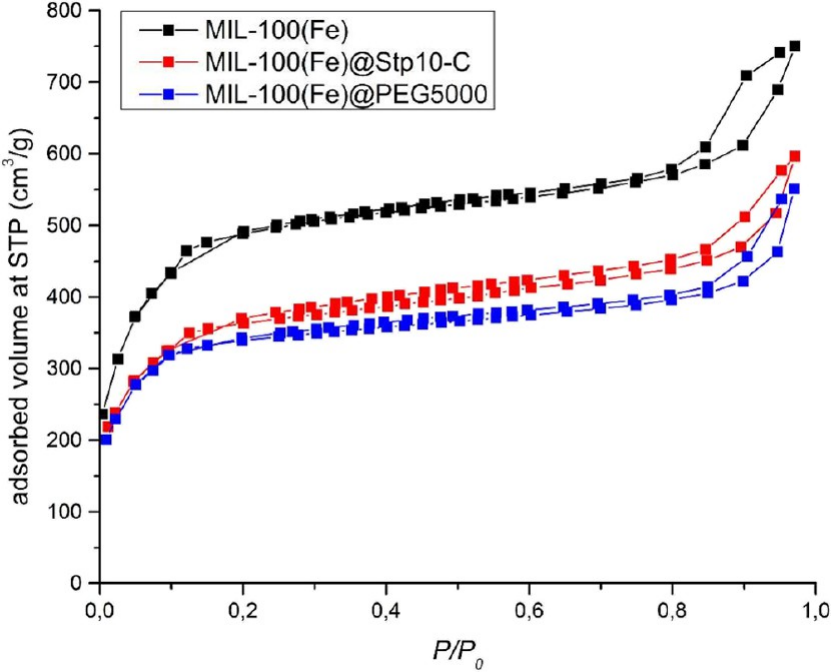
N_2_ adsorption at 77K of coated and uncoated MIL-100(Fe) nanoparticles. Reprinted with permission from ref (82). Copyright 2016 American Chemical Society.

**Figure 16:**
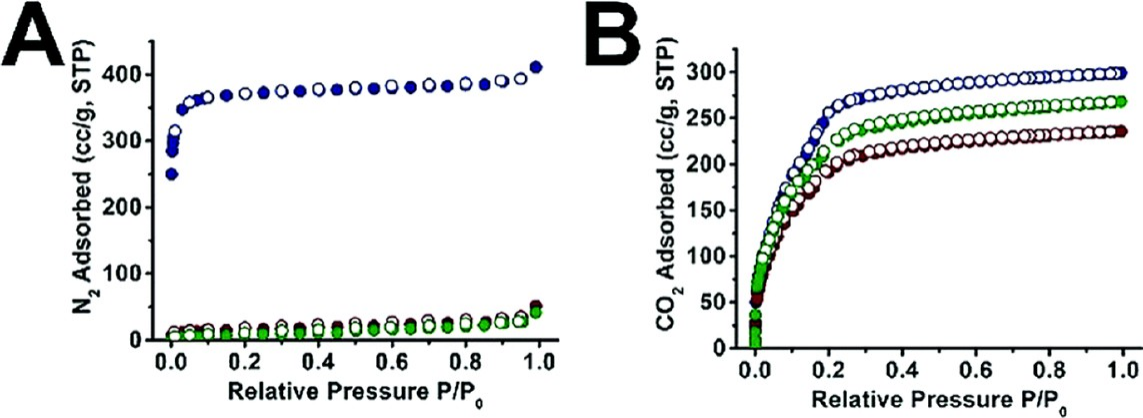
(a) N_2_ sorption isotherms at 77 K, (b) CO_2_ sorption isotherms at 195 K. UiO-66 (navy), UiO-66@xPS (red) and UiO-66@xPBA (green). Adapted with permission from ref (126). Copyright 2019 Royal Society of Chemistry.

Not only does grafting density affect membrane gas separation and mechanical properties, it also affects gas transport through the coating itself. For example, when comparing two PDMS-coated MOFs, one coated via a *grafting from* mechanism^127^ and the other via *grafting to*,^84^ the former blocks cryogenic N_2_ adsorption, while the latter allows it to occur. This difference is likely due to the denser coating achieved through the *grafting from* approach. This phenomenon is further illustrated with a comparison of adsorption isotherms attained from two classes of aforementioned PEFs. In the first set of materials, the poly(*n*-butylacrylate) (PBA) and poly(styrene) (PSty) coatings are reported to have a grafting density of 5.3 initiator molecules per nm^2^ and a coating thickness of 32 nm. The second set of materials based on PEG has a grafting density of 0.1 molecules per nm^2^ and did not report a coating thickness, although the TEM images appear to show thickness between 10 and 20 nm. We acknowledge that these coatings are comprised of different monomers, but the N_2_ adsorption for all these coatings were performed at 77 K, well below the *T*_g_ of all these polymer coatings. Polymer segmental motion as well as chain end motion are known to facilitate gas diffusion through amorphous polymers; thus, diffusion will be dramatically slowed when the polymer is in a glassy state at these cryogenic temperatures. The N_2_ adsorption isotherms for the PEF with a thin, low-density coating is shown in Figure 15. The decrease in adsorption can largely be attributed to the increased sample mass of the coating. However, it can be observed in Figure 16 that when the coating is much more dense, the coated material does not uptake any N_2_, though it can uptake CO_2_ when warmed an additional 120 degrees. The results imply that the density and thickness of the coatings can dramatically influence gas permeation in these materials, which becomes pronounced at lower temperatures.

The above results suggest that it is possible to kinetically trap adsorbed gas inside the pores of the PEF by lowering the temperature of the material after adsorption has occurred, effectively slowing or preventing diffusion through the polymer shell. This phenomenon was recently reported for a PDMS-coated COF material which demonstrated kinetic trapping of H_2_ which increased the temperature of desorption of H_2_ (vide supra).^41^ This idea remains largely unexplored for gas storage applications.

The grafting density of a polymer coating onto a nanoparticle surface can vary depending on the coating method. Several works in this review calculate grafting density, allowing for useful comparisons. Covalent-driven *grafting to* resulted in a low density of about 0.1 polymer chains per nm^2^. In the grafting from technique, a much higher density of 5.3 initiator molecules per nm^2^ could be achieved, followed by SI-ATRP.^82,126^ The number of initiator molecules per nm^2^ may not directly reflect the final grafting density, as not every initiator may result in a fully formed polymer chain. However, this data suggests the *grafting from* method can increase grafting density by more than an order of magnitude. This significant difference between *grafting to* vs *from* is consistent with broader literature observations.^151^ Notably, postsynthetic exchange allylation of UiO-66 followed by hydrosilylation with PDMS achieved a high density of approximately 1.5 chains per nm^2^, surprisingly high for a *grafting to* mechanism.^84^ GraftFast, a method involving chemisorption of aryl radicals for initiating covalent attachment of polymers, resulted in densities ranging from 0.05 to 0.9 chains per nm^2^, the latter value being relatively high for a *grafting to* mechanism.^87,88^ These two *grafting to* mechanisms suggests that introducing reactive sites to the particle surface before polymer attachment can enhance grafting density of this mechanism. In a thiol-functionalized *grafting to* method, the grafting density ranged from 0.6 to 0.9 chains per nm^2^, with a decreasing molecular weight of the polymer.^104,105^ This suggests an advantage of the metal CUS attachment mechanism.

Control over coating thickness, density, and surface-exclusion are important for maintaining the integrity and functionality of these composite materials in gas storage and separation applications. Our analysis indicates that higher grafting densities are more suitable for gas storage and durability applications, where a dense and less permeable coating is preferred. Low to moderate grafting density may be more advantageous when cost or processability are of primary concern. Examples of different coating methods and their resulting thicknesses and grafting densities are summarized in Table 1. In the next section, we delve into the challenges associated with characterizing PEFs, further highlighting the complexities as well as potential advantages of these materials in the field.

**Table 1:** PEF Coating Methods and Representative Examples of Resulting Thicknesses and Grafting Densities

Coating method	Polymer	Grafting Density	Thickness	Reference
**Coordinative grafting to**	RAFT copolymer based on PNIPAM	0.6–0.9 chains/nm^2^	2.5–10 nm	(104,105)
**Covalent grafting to**	PEG	0.05–0.9 chains/nm^2^	nm scale	(87,88)
**Covalent grafting to**	PDMS	1.5 chains/nm^2^		(84)
**Covalent grafting to**	PEG, Stp10-C	0.1 chains/nm^2^		(82)
**Covalent grafting from**	PSy, PBA	5.3 initiator/nm^2^	13–78 nm	(126)
**Weak IMF grafting from**	Poly(2-hydroxyethyl methacrylate), poly(2,2,2-trifluoroethyl methacrylate) and poly(3-methacryloxypropyltrimethoxysilane)		15–85 nm	(129,130)
**Thermal vapor deposition**	PDMS		10–15 nm	(137,138)
**Nonsolvent-induced coatings**	Polyimide, polysulfone		5–20 nm	(131,132)

## 3 Challenges Associated with Characterization of Coated Framework Materials

Given the wide range of coating techniques and diverse PEF materials documented in the literature, meaningful comparisons of gas sorption data sets across different research groups can only be attained if a comprehensive characterization and quantitative assessment of the materials was conducted. It is important that these characterization techniques verify the presence and integrity of the coating, confirm the preservation of critical framework characteristics, and ascertain bond formation between polymer and framework before drawing specific conclusions. While numerous quantitative characterization methods like powder X-ray diffraction (XRD), nuclear magnetic resonance spectroscopy (NMR), and Fourier-transform infrared spectroscopy (FTIR) are routinely used in the literature, and are thus not reviewed here, other seemingly routine techniques may pose challenges during characterization due to the heterogeneous nature of the hybrid materials and difficulties in analyzing materials with soft, flexible organic polymer shells and rigid, inorganic crystalline framework cores.^152−155^

In this context, we examine challenges related to surface area analysis of PEF materials using gas adsorption isotherms. We discuss electron microscopy techniques, such as transmission electron microscopy (TEM) and scanning electron microscopy (SEM), along with dynamic light scattering, as methods to discern coating characteristics in PEF materials. Additionally, we offer our perspective on some powerful yet underutilized techniques that could provide insight into material characteristics responsible for promoting and dictating gas diffusion in PEF materials, including broadband dielectric spectroscopy and various fluorescence techniques.

### 3.1 Effect of Coating on Surface Area

Understanding the changes in surface area of materials before and after coating is crucial for predicting their performance in certain applications. However, conventional techniques for characterizing surface area through gas adsorption may not provide a clear indication of whether the pores in a coated framework are blocked by polymers, solvents, or other contaminants, or if the coating simply inhibits gas permeation. Surface areas are commonly evaluated by measuring N_2_ adsorption isotherms at 77 K and applying the Brunauer–Emmett–Teller (BET) theory to calculate the surface area. However, this approach is contingent on the ability of N_2_ to permeate the polymer coating at cryogenic temperatures on the time-scale of the BET measurement. At these low temperatures, most polymers are in a glassy state, which greatly hinders gas permeation (see discussion below on polymer mobility). Determining whether the impediment to N_2_ adsorption is a thick, dense polymer coating or pore blockage requires additional analysis. In scenarios where the polymer coating remains flexible at ambient temperatures, adsorption isotherms utilizing CO_2_ or water vapor can be performed (Figure 16). This allows for the comparison of isotherms between coated and uncoated samples. While CO_2_ is not ideal for deriving absolute surface areas in BET assessments due to notable CO_2_–CO_2_ interactions, it serves as a useful proxy for understanding the comparative porosity and structural changes in the material’s framework before and after the application of a coating.^156^

Indeed, many of the studies reviewed above incorporated N_2_ adsorption and surface area analyses for coated materials into the characterization. Some findings revealed that the porosity and surface area of the framework materials were largely preserved postcoating,^74,75,79,87−90,118,122,123,126,127,130−132,138^ suggesting that the polymer coatings were not sufficiently dense to prevent N_2_ penetration, even at temperatures where the polymers were glassy. Conversely, other studies reported a notable reduction in surface area following the coating process.^78,83,93,96,103,124,128^ While pore volumes are quantifiable in certain instances, challenges arise when the quantity of N_2_ adsorbed is insufficient for precise pore volume measurements. In such scenarios, employing CO_2_ adsorption isotherms at near-ambient temperatures emerges as a valuable supplemental tool for material characterization.^41,78,83,93,96,103,124,128^ If the adsorption of N_2_ is impeded by the dense, glassy coating at 77 K, CO_2_ may still be adsorbed at near-ambient temperatures where the coating becomes more permeable. If N_2_ adsorption is prevented due to the pores being filled or clogged, CO_2_ adsorption will likely be hindered as well. Therefore, employing this method alongside N_2_ adsorption can help identify the reasons behind diminished surface area. Other porosity measurement techniques which do not require cryogenic temperatures include mercury intrusion porosimetry, positron annihilation lifetime spectroscopy (PALS), and X-ray scattering. Mercury intrusion characterizes nanopores above 3 nm to macropores up to 400 μm, but for pores below this size it is not as applicable.^157,158^ Small-angle X-ray scattering (SAXS) is a powerful technique to characterize nanopore structure, allowing determination of surface area as well as pore connectivity.^159−161^ SAXS is also a faster measurement than gas adsorption isotherms, which is advantageous. PALS is another technique which can be used to characterize micro- and meso-pores without need for a probe molecule.^162−164^ This technique is able to detect open and closed pores, which is an advantage. Metal ions in MOFs can be sources of interference, however. There are several alternatives to gas adsorption isotherms at cryogenic temperatures, but most are best done in parallel to gas adsorption as the latter is the most practical way to characterize the porosity of framework materials, which have pores ranging from micro- to mesopores.

### 3.2 Coating Thickness and Particle Size Distribution

**Figure 17:**
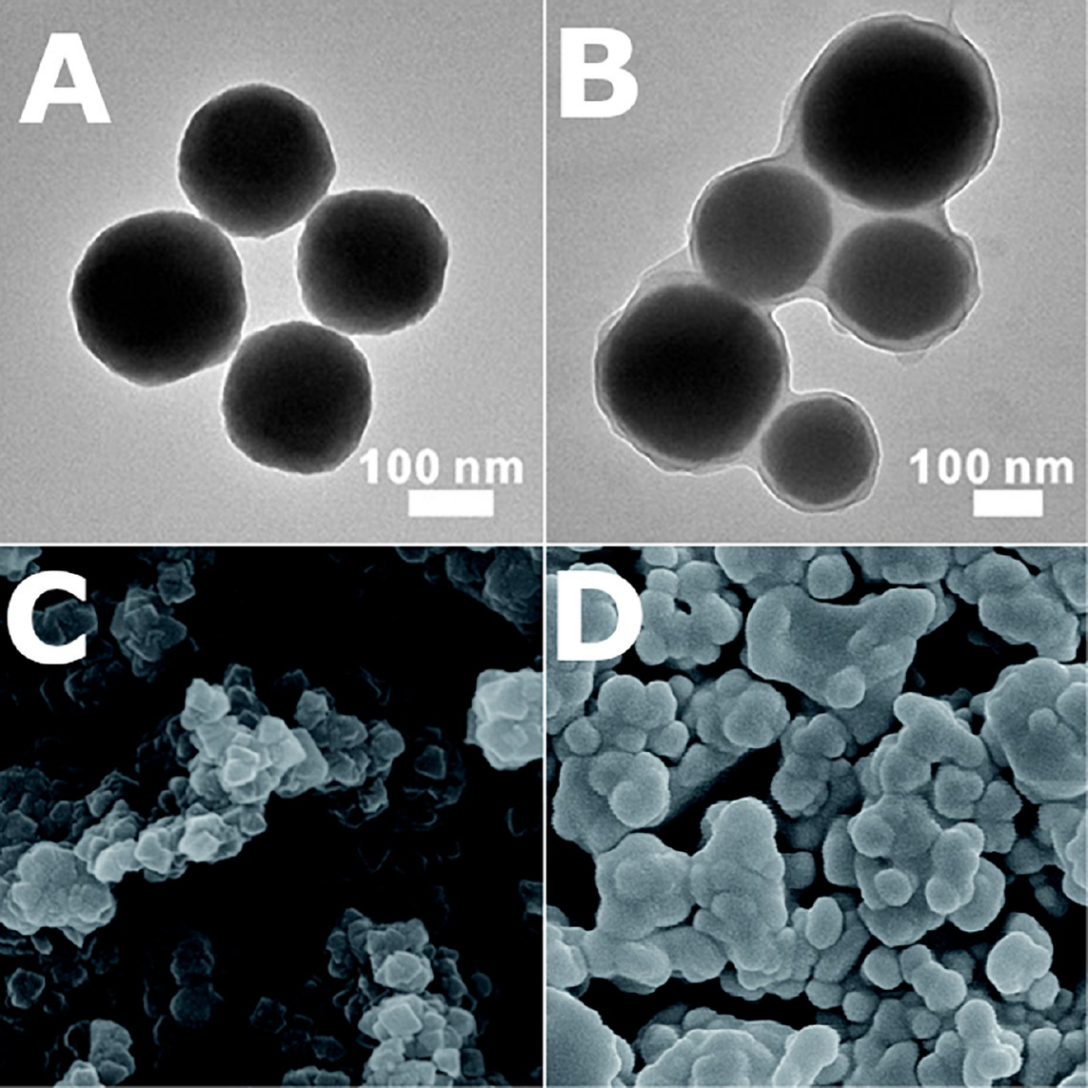
(a) TEM images of a MOF and (b) a PEF showing the distinct coating visible on the edges of the particles. Adapted with permission from ref (129). Copyright 2021 American Chemical Society. (c) Field emission-SEM (FESEM) images showing UiO-66-NH_2_ and (d) coated PMMA-*g*-GMA-UiO66-NH_2_ showing a size increase after coating. Adapted with permission from ref (93). Copyright 2018 Royal Society of Chemistry.

Transmission electron microscopy (TEM), scanning electron microscopy (SEM), and scanning transmission electron microscopy (STEM) can be very useful for characterizing both the coating thickness in PEFs, as well as the particle size distribution of the framework materials before and after the coating process. Images resulting from the interactions between electrons and materials are influenced by the specific location, surface features, and the elemental composition encountered by each electron in the beam.^165^ Therefore, MOFs and polymers can often be differentiated in electron microscopy images, as metals interact very differently than organic elements with the electron beam. However, organic materials are particularly vulnerable to degradation in a strong electron beam. Mitigating material degradation requires either reducing the beam’s intensity or reducing the exposure time, both of which can compromise the signal-to-noise ratio. Despite these challenges, numerous studies have successfully utilized electron microscopy to unambiguously demonstrate the formation of distinct coatings in these materials (Figure 17 a–b).^41,74,75,79,104,118,119,123,126,129,131,132^ In other instances, the resultant images less clearly reveal the coatings, showing only variations in size or morphology (Figure 17 c–d).^76,83−85,87−94,96,103,107,115,116,120−122,125,130,136^ The latter is particularly common for COFs when both the polymer and framework are composed entirely of “soft” elements and interact with the beam in a similar manner.

**Figure 18:**
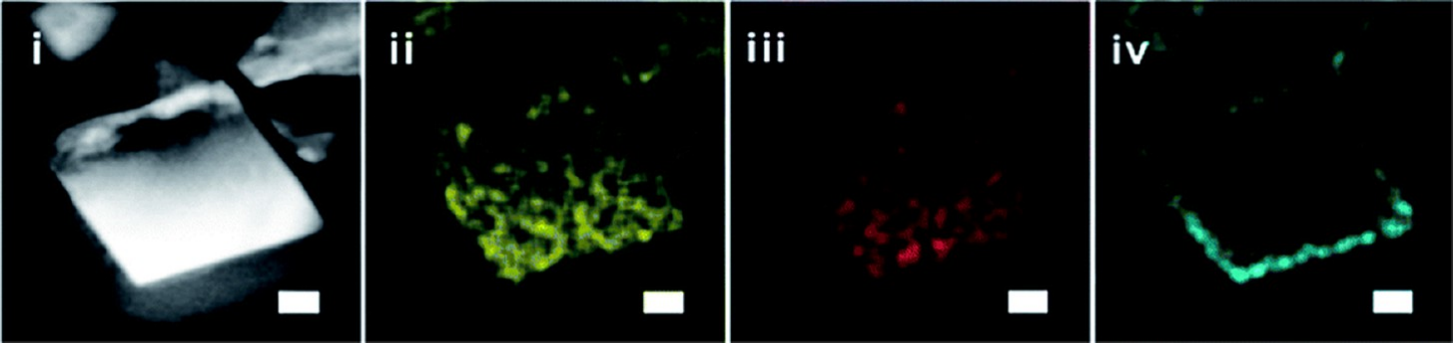
(i) STEM image of UiO-66@polymer and EDS mapping of (ii) Zr, (iii) O, and (iv) Cl showing the layer of Cl on the surface of the particle. Scale bars = 100 nm. Adapted with permission from ref (126). Copyright 2019 Royal Society of Chemistry.

While SEM and TEM techniques do not always show a clearly defined coating due to agglomeration or component similarity, the complementary technique of energy-dispersive X-ray spectroscopy (EDS) can aid in distinguishing the coating from the framework in certain materials. In coated MOFs and COFs where a distinct element is present in the polymer coating, EDS can be very useful in helping to verify whether the coating penetrates the pores or is present largely as a core–shell structure. EDS analyzes the characteristic X-rays emitted when an electron beam interacts with the sample during electron microscopy, mapping the element-specific signals to specific positions on the sample.^166^ This process generates an elemental map of the sample, as depicted in Figure 18. For MOFs, the metal atom present in the framework can be the distinctive element. If the coating is genuinely surface-excluded, the particle edges will exhibit a higher concentration of the signal corresponding to the coating (and *vice versa* if the unique element is in the core). EDS analysis has been instrumental in characterizing many of the coatings in literature PEF materials.^41,111,119,123,124,126,127,129,132,136−138^ TEM-EDS techniques typically reach 1–20 nm spatial resolution, while STEM-EDS reaches ∼1 nm resolution or below (even to angstrom scale) depending on sample thickness.^167,168^ This range makes this technique very applicable for identifying nanometer scale coatings on nanoparticles.

Dynamic light scattering (DLS) can also serve as a valuable tool for estimating changes in particle size pre- and postcoating, from which coating thickness can be inferred. However, caution is warranted in interpreting the results. While polymer coatings will generally increase particle size, which will be accompanied by a rise in average hydrodynamic radius of the particle, discrepancies can arise. For instance, He et al. found in their study on porous liquids synthesized from coated MOFs that an increase in DLS hydrodynamic radius did not align with estimates from SEM data.^127^ They attributed this inconsistency to swelling induced in the polymer by the dichloromethane solvent. Introducing a small amount of poor solvent (methanol) to the solution led to a reduction in observed hydrodynamic radius, better aligning it with SEM-derived particle size. Furthermore, aggregation induced by poor solvents can artificially inflate the average particle size recorded in these measurements. Therefore, while informative, DLS should be viewed as a complementary characterization technique in determining coating thickness.

Other structural characterization techniques include SAXS, and small-angle neutron scattering (SANS). SAXS and SANS are powerful techniques which allows determination of particle size and radius of gyration, structural differences and interactions between core and shell, and coating properties such as thickness, heterogeneity, grafting density, and thickness variation. SAXS is an X-ray scattering technique which results in higher signal than neutron scattering, and typically is more accessible than SANS experiments.^169−173^ However, SAXS is generally only a useful probe of the properties of (semi)crystalline materials. Neutrons can penetrate matter further than X-rays, are isotope-sensitive, and provide information about amorphous materials. As such, SANS can give even more information about a nanocomposite material than SAXS, although it requires longer data acquisition times and is a less accessible technique.^174−180^

### 3.3 Polymer Mobility

Polymer mobility refers to the ability of polymer chains to move or reorient themselves within a material. This characteristic is crucial in understanding the transport properties of gases through polymeric materials, as the ease with which polymer chains can move affects the material’s permeability to gases.^181^ According to the free volume theory, gas diffusion and polymer mobility are intricately linked, where gas diffusion occurs through transient free volume elements in a polymer matrix that arise due to the movement of polymer chains.^182^ Increased mobility leads to a higher frequency and size of these free volume elements, which in turn facilitates the diffusion. The relationship is complex, as factors such as temperature that increase mobility can also cause the polymer to swell, affecting gas solubility and, in turn, the overall permeation rate. However, understanding mobility plays an important role in the optimization of PEF materials for a given application.

While conventional techniques like viscometry and thermal or dynamic mechanical analysis are commonly employed to investigate mobility in bulk polymer systems, these techniques are less effective for studying the mobility of surface bound polymers in PEF materials. Differential scanning calorimetry (DSC) is a widely used technique that measures changes in heat flow associated with transitions in a polymer, such as the glass transition temperature (*T*_g_), which is directly related to polymer mobility.^183^ Above *T*_g_, polymer chains are said to be rubbery, and polymer segmental mobility (motion associated with ∼50 carbon-sized atoms) increases, facilitating enhanced gas diffusion. However, DSC is sometimes not sensitive enough to determine thermal transitions in thin films or at low polymer loadings in PEF materials or polymer-composites. Furthermore, the independent mobility of side chains and chain ends that occur at temperatures below the glass transition, known as sub-*T*_g_ motions, also play a role in promoting diffusion processes.^184^ The latter motions are believed to facilitate aging phenomenon in glassy polymers,^185^ and have also been invoked to explain gas diffusion into glassy polymers as a function of that aging.^186^ These subsegmental motions occur on a fast time scale, while the segmental motions associated with *T*_g_ and other phase transitions such as melting point occur on longer time scales.^187−189^ DSC is limited to detecting thermal transitions at longer time scales and can be signal-to-noise limited, particularly for thin films or coatings with broad phase transitions. Thus, DSC is not always an ideal technique for analyzing thinly coated PEFs or their gas diffusion properties.

Recent developments in fluorescence techniques offer some intriguing opportunities for characterizing thermal transitions and mobility in thinly coated PEF systems.^190^ Given the sensitivity of specific fluorescent probes to the rigidity of their matrix, as evidenced by changes in the shape of the emission spectra and the overall emission intensity,^191^ these probes have been effectively employed to identify thermal transitions in bulk polymers.^192,193^ However, the utility of these fluorescent methods extends beyond this application as they are notably adept at analyzing thermal transitions in thin films and nanocomposites, which as discussed can be challenging to discern through conventional means.^193−195^ An additional key advantage of these methods is the ability to place or tether probe molecules at specific sites within the polymer structure or system architecture. The technique(s) have proven particularly useful for examining mobility in layered films,^196,197^ at the interfaces between different polymers,^198,199^ and for correlating polymer mobility with gas diffusion processes.^200^

Broadband dielectric spectroscopy (BDS) can provide additional insight into polymer mobility and subsequent diffusion phenomena by analyzing the movement of electronic dipoles in a material across a broad range of suboptical frequencies, from 10^–5^ to 10^12^ Hertz.^201,202^ A unique feature of BDS that distinguishes the technique from fluorescence and other traditional methods for investigating thermal transitions in polymers is its nuanced ability to differentiate between the segmental motion of polymers, which involves large groups of carbon atoms and occurs at longer time scales, and the more localized independent movements of side chains and chain ends which occur on shorter time scales.^187−189,203^ These latter movements, occurring at temperatures below the glass transition temperature (sub-*T*_g_ motions), are believed to be responsible for facilitating the slow gas diffusion that occurs in glassy systems. Additionally, BDS is highly sensitive, with nanometer-scale films and even single polymer molecules able to be studied through the technique.^204−206^ As BDS detects fluctuation of dipole moments or charge movement in a material, and not molecular motion directly, assigning mobility detected by BDS to site-specific molecular motions requires additional insight from complementary techniques such as NMR.^207^ NMR relaxometry is a powerful technique to analyze polymer mobility, as it is able to detect molecular motions from 10^–6^ to 1 s time scale with the advantage of site-specificity.^208−210^ Spin–spin and spin–lattice relaxometry allow for the determination of specific molecular motions in polymers from subsegmental to bulk.^211−214^ Paired with a broader technique like BDS, the detection and assignation of a wide range of molecular motions is possible, allowing greater understanding of polymer dynamic motion.

The intricate relationship between polymer mobility and gas diffusion underscores the importance of selecting and applying the most suitable analytical techniques to understand and optimize these processes in PEF materials. Despite being underutilized for the study of PEFs, techniques like fluorescence spectroscopy and BDS, especially when used in conjunction with NMR, could provide comprehensive insights into the dynamic behavior of polymers at various scales. Such insights would be crucial for fine-tuning the properties of PEF materials to meet specific application requirements, ultimately enhancing their performance in gas storage and separation applications.

## 4 Summary of Advances Facilitated by PEFs

PEFs improve the processability and durability of framework materials, resulting in exciting material advances, including extremely flexible and durable MMMs and ultrastable MOFs, to practical achievements in advancing gas storage and separation capabilities.

### 4.1 Advances in Gas Storage and Separation

Some of the most significant advances discussed in this review relate to improvements in gas storage and separation technology facilitated by PEFs. PEFs have sparked exciting developments in gas storage technology, such as the ability of a PDMS-bottlebrush coated COF to kinetically trap H_2_ at temperatures (below the *T*_g_ of the polymer) where the coating was glassy and impermeable.^41^ This is a novel method of increasing the temperature at which it is possible to store H_2_ in a sorbent, a critical aspect of practical H_2_ storage. Additionally, PEFs offer stability improvements mentioned vide infra, that are beneficial for handling humid gas streams and ensuring atmospheric stability.

**Figure 19:**
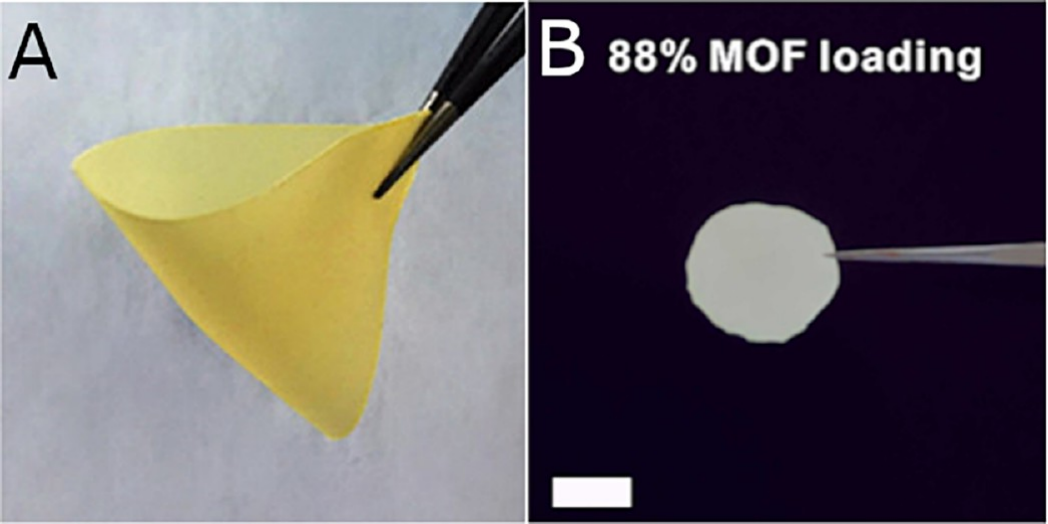
Highly flexible freestanding MMMs at very high MOF loadings are possible with polymer-coated MOFs as the porous filler. (a) Flexibility of MMM formed via ROMP grafting through. Adapted with permission from ref (94). Copyright 2018 American Chemical Society. (b) Freestanding MMM at 88% MOF loading via covalently grafted polyimide brushes to form PEFs, scale bar = 5 mm. Adapted with permission from ref (118). Copyright 2018 American Chemical Society.

Another greatly impactful category of advancement is in utilizing PEFs over pristine MOFs as porous fillers in MMMs for gas separation. Although MMMs are very promising for gas separation, defects can arise where the crystalline inorganic framework and flexible polymer chains are incompatible, leading to strained polymer chains and issues like void formation, pore blocking, and membrane rigidification.^11,32,215−217^ These all reduce permselectivity of the membrane. PEFs have emerged as a solution to these challenges. Through high compatibility between the polymer-coated framework surface and the bulk polymer, PEFs significantly reduce interfacial defects, reduce aggregation, and increase dispersibility, resulting in improved membrane properties (Figure 19).^84,88,93,118^

For example, covalently grafted polyimide brushes on the surface of a MOF resulted in a nearly 5-fold increase in the tensile and shear strength, decreased the rate of aging and defect formation, and greatly increased the permselectivity of CO_2_/N_2_ and CO_2_/CH_4_ as compared to the uncoated MOF MMM (up to a 60% increase).^118^ Additionally, a PDMS brush-grafted MOF formed MMMs displayed a 1.4-fold increase in mechanical strength over the uncoated MOF MMM, while increasing CO_2_/N_2_ selectivity and larger molecule size-sieving ability.^84^ PEFs as porous fillers in MMMs have resulted in highly interfacial, durable, and selective membranes.

Several PEFs discussed here contribute to another development in MMM technology: *in situ* polymerization of MMMs.^85,90,93,94,96,116,118^*In situ* polymerization creates an integrated membrane, maximizing interfacial interaction and resulting in improved mechanical durability and gas separation selectivity in the resulting network polymer membrane. For instance, a polynorborene-MOF membrane displayed a 170% increase in tensile strength compared to the ungrafted MOF MMM.^94^ Additionally, a MOF functionalized with GMA before *in situ* polymerization of MMA to form a PMMA-MOF membrane demonstrated a significant increase in mechanical strength while improving membrane selectivity for CO_2_/N_2_, CO_2_/CH_4_, as well as He/N_2_ and He/CH_4_.^93^ PEFs and the concept of surface-excluded polymerization have led to notable developments in MMM technology, significantly improving critical aspects of MMM materials.

### 4.2 Improved Framework Form Factors

One of the interesting and broadly applicable aspects to PEFs is the variety and flexibility of form factors in the resulting materials. Through various synthetic methods, researchers have successfully produced PEFs ranging from hydrophobic solids to porous liquids, as outlined in Table 2. Among the discussed PEFs, a significant emphasis has been placed on producing hydrophobic MOF solids, representing the largest category of form factors.

**Table 2:** Summary of Form Factors Resulting from Various Coating Mechanisms and Methods

Form factor	Coating mechanism	Coating method
Hydrophobic nanoparticles	Coordinative	Coordinative surface PSE with polymer,^107^*grafting to* metal CUS^104−106^
	Covalent	GraftFast,^87^ click modulator PSM,^89^ surface ligand *grafting to*,^82,83^ pendant moiety ATRP initiator attachment,^77,78^ pendant moiety RAFT CTA attachment,^111^ polymer@MOF@MOF,^79^ PMOFSA,^86^*grafting through* pendant NH_2_ moiety^92^
	H-bond	Self-assembly of initiator on MOF surface^126^
	Weak IMF	TVD,^136−138^ solution-based *grafting to,*(119−122,125,130) thermal cross-linking after polymer adsorption,^123,124^ site-specific free radical source,^81^ polymerization in solution,^128^ NISAP,^131^ NISD^132^
Pellet	Weak IMF	NISD^132^
Suspension	Covalent	Grafting to pendant NH_2_ moiety,^76,115^*grafting through* pendant NH_2_ moiety^91^
Porous liquid	H-bond	Self-assembly of initiator on MOF surface^127^
	Covalent	Surface ligand ATRP initiator attachment^41^
Membrane^a^	Coordinative	ATRP initiator coordinative surface PSE,^74^ ATRP initiator bound to metal CUS^103^
	Covalent	RAFT CTA attachment to metal CUS,^75^ click reaction *grafting through,*(85)*grafting through,*(93,94,116) step-growth *grafting through*(96,118)
	H-bond	PISA^129^
	Weak IMF	Solution-based photopolymerization *grafting through*(90)
MMM	Covalent	Covalent bond to pendant NH_2_ moiety,^93,118^ covalent *grafting to* after PSE allylation,^84^ Graftfast^88^

a Integrated membranes which consist solely of PEFs, whether through polymer-driven self-assembly or *in situ, grafting through* polymerization of the membrane.

**Figure 20:**
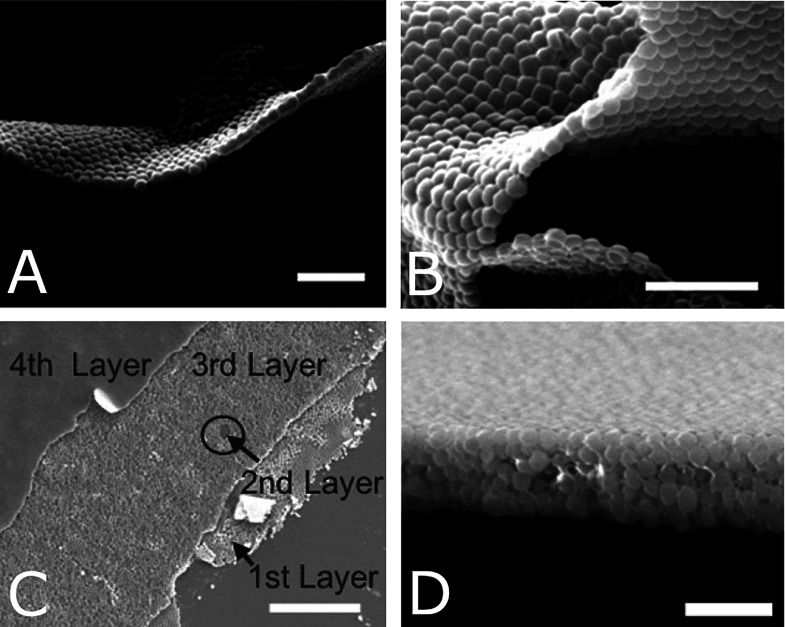
SEM images of self-assembled membranes of coated MOFs: (a–b) monolayer films, scale bars = 1 μm, (c) self-supporting multilayer film, scale bar = 5 μm, (d) cross-section of multilayer film, scale bar = 1 μm. Adapted with permission from ref (74). Copyright 2019 American Chemical Society.

Thin film and single-component membranes are another key development in the MOF membrane space. Cohen and colleagues successfully produced self-assembled multilayer and monolayer thin film membranes from PEFs (Figure 20).^74,75^ These monolayer thin films were closely packed with few defects. Li et al. also achieved a monolayer, self-supporting 200 nm thick film.^129^ These self-supporting, single-component films have advanced the field of MOF membranes by eliminating the need for a matrix polymer, allowing for maximum MOF loading. The highly flexible and durable monolayers address an issue in gas separation membrane technology whereby high loadings result in brittleness and interfacial incompatibility. This development has introduced a new class of membranes with great potential for gas separation applications.

Porous liquids have been achieved via PEFs as well. Coated MOFs formed permanently porous liquids via colloidal suspensions in PDMS,^127^ as well as coated COFs in PEG and PDMS.^41^ The coatings efficiently exclude the solvent/matrix polymer from the pores of the framework, resulting in a permanently suspended porous liquid. Porous liquids have garnered significant attention in the realm of gas storage and separation applications, as they can be handled and transported like conventional liquids. In addition to the advantages in processability for liquids, they have the potential to utilize existing infrastructure for liquid-based storage and separations. Innovative form factors could lead to progress in the efficiency of gas storage and separation, with those achieved by PEFs offering improved gas transport, increased loading capacity, and compatibility with existing infrastructure.

### 4.3 Improvements in Chemical and Mechanical Stability

**Figure 21:**
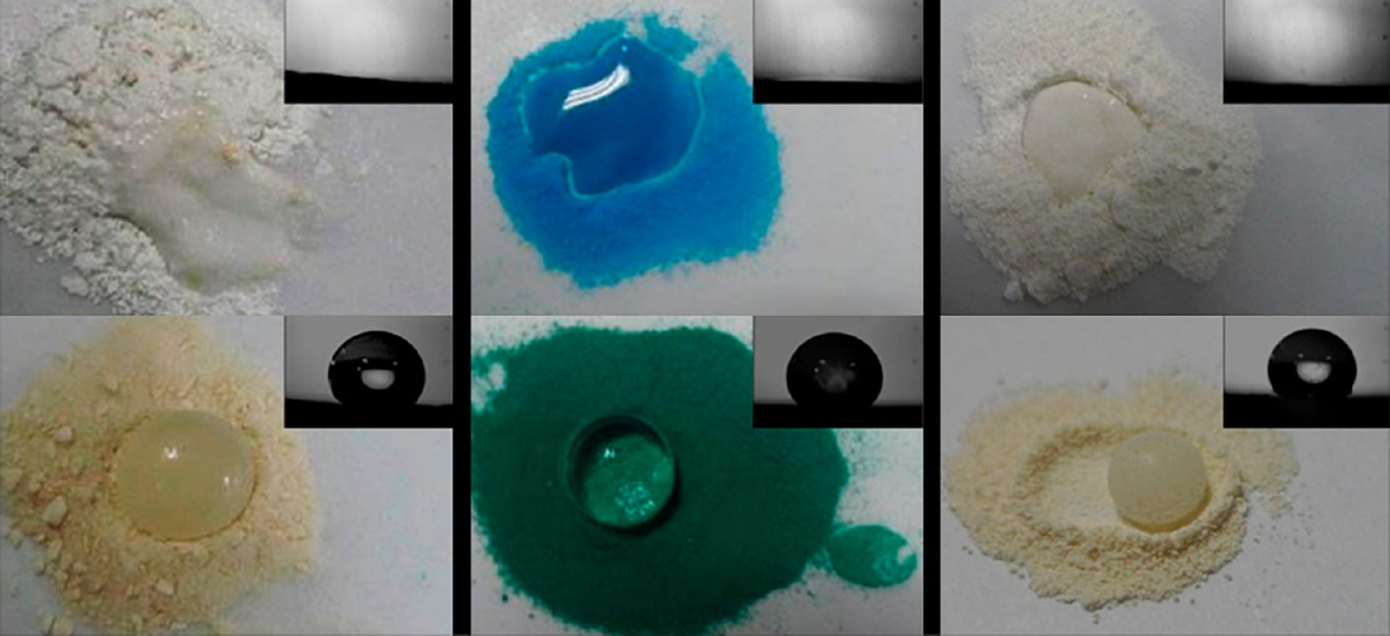
Water contact angle measurements of MOFs before (top) and after (bottom) polymer coating. Adapted with permission from ref (136). Copyright 2014 American Chemical Society.

PEFs offer remarkable improvements in stability compared to their pristine counterparts, ranging from resistance to moisture and harsh chemical environments to improved mechanical durability. The coatings can have an impact on dispersibility and stability in different solvents as well. These enhancements make PEFs highly promising for gas storage and separation applications. In scenarios where humidity is a concern, such as in storing water electrolysis-derived hydrogen or separating flue gas emissions, and in all applications exposed to ambient conditions, PEFs hold a distinct advantage. Uncoated framework materials can decompose or experience irreversible damage upon exposure to water. Many of the PEFs discussed in this review exhibit substantial improvements in hydrolytic stability and water resistance, as demonstrated by water contact angle measurements in Figure 21.^79,80,119,120,122,124,126,132,136,137^

These coated frameworks can withstand humidity and water immersion without suffering a loss of crystallinity or surface area. Certain PEFs maintained CO_2_ adsorption capacity after exposure to water or humidity, a promising result for improved cyclability in humid conditions.^119,120,124,136^ Additionally, H_2_O adsorption and humid CO_2_ adsorption were measured in some PEFs, indicating the coated materials’ high resistance to hydrolytic decomposition even when water molecules are adsorbed into the pores of the structure.^120,124,126,129,137^ This progress broadens the range of applications for frameworks in situations where humidity is a factor.

**Figure 22:**
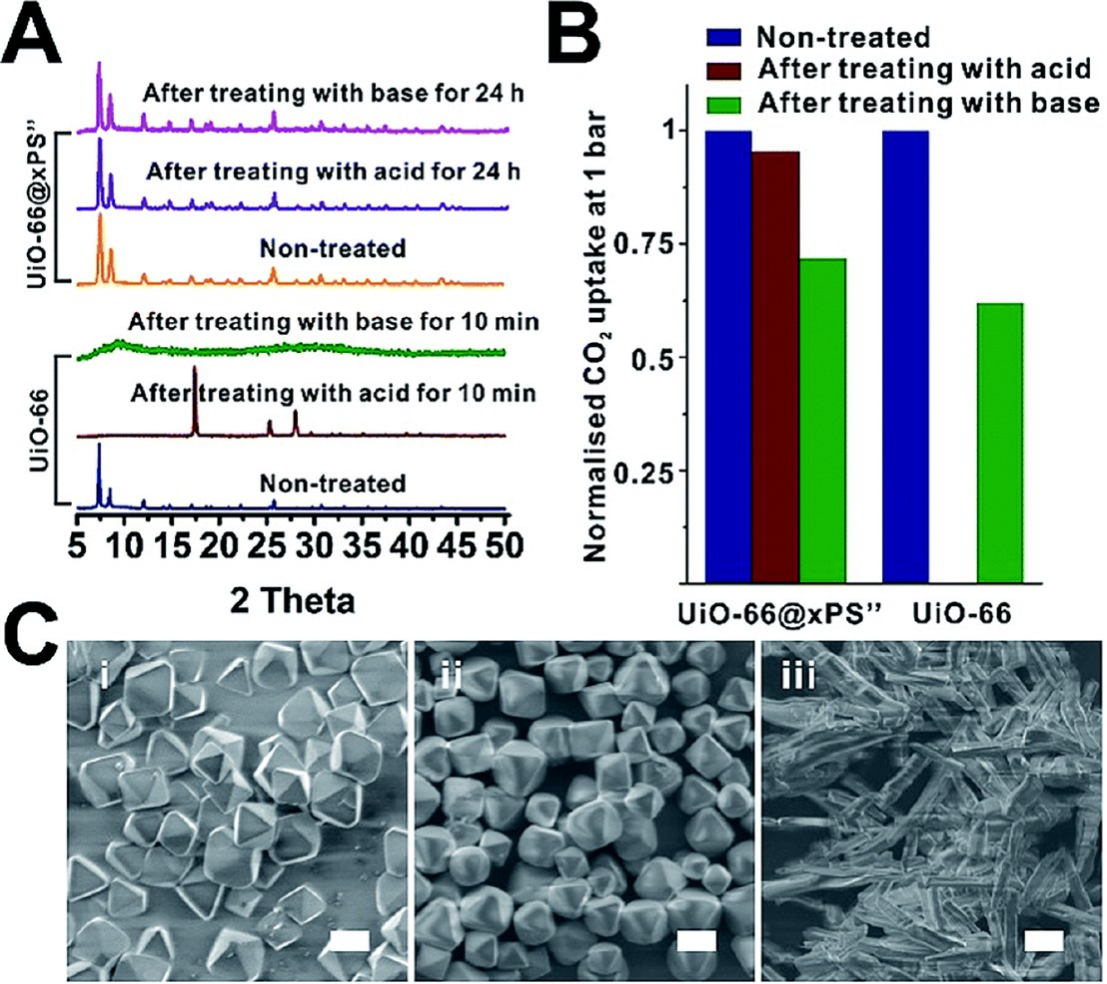
(a) PXRD patterns and (b) normalized CO_2_ uptake of untreated MOFs and PEFs and after exposure to strong acid or base. (c) SEM images of i. MOFs, ii. PEFs after 24 h acid treatment, and iii. MOFs after 10 min acid treatment. Scale bars = 1 μm for i. and ii., and 2 μm for iii. Reprinted with permission from ref (126). Copyright 2019 Royal Society of Chemistry.

The stability conferred by polymer coatings extends to harsh chemical environments as well. Specific coatings have resulted in MOFs that remain stable even when exposed to strong bases like NaOH or strong acids such as H_2_SO_4_, even after prolonged exposure at high concentrations.^126,132^ As shown in Figure 22, coated MOFs retain their crystallinity, morphology, and porosity, while pristine MOFs subjected to the same corrosive conditions degrade significantly.

PEFs can improve the colloidal stability of framework materials in solvents. Dispersibility of framework materials depends on their polarity relative to the suspending solvent. Polymer coatings can bring about changes in the dispersion behavior due to the formation of what is typically a nonpolar polymer shell over a polar framework material core. As a result, enhanced dispersibility and colloidal stability in various solvents is often observed for coated materials, including water, which causes many noncoated framework materials to irreversibly aggregate and degrade. PEFs have also been shown to enhance the mechanical stability of MOFs, particularly with respect to pelletization. Coated MOF nanoparticles facilitate pellet formation with significantly reduced fracturing and defect formation.^132^ Overall, polymer coatings significantly enhance the stability of framework materials, whether in the presence of moisture, harsh chemicals, or mechanical stress. The improvement in form and function that coated framework materials show over pristine frameworks is exceptional.

## 5 Conclusions and Outlook

This review has provided a comprehensive overview of polymer-encapsulated framework materials (PEFs) and their significant impact on framework material stability and usability in gas storage and separation applications. The improved processability and durability offered by these coatings could facilitate the implementation of framework materials commercially. PEFs have also led to diverse form factors, such as flexible monolayer membranes, porous liquids, and hydrophobic powders, which allow for possible applications where traditional microcrystalline powders may not be the ideal choice. PEFs have already driven significant advancements in the domain of gas storage and separation, with a particular focus on enhancing processability and stability, two key implementation challenges. PEFs significantly improve on concerns regarding the mechanical pressing of MOFs and COFs for separations. Polymer coatings have also transformed the chemical stability of framework materials, especially hydrolytic stability. Furthermore, PEFs have greatly improved membrane technologies. These advances directly address issues that currently affect implementation of framework materials in industrial gas separation.

Despite the successful creations of PEFs discussed so far, challenges and opportunities for future research remain. One key challenge is that many of the effective coating methods are resource-intensive, involving the synthesis of custom molecules and time-consuming polymerization processes. Simplified coating methods such as PMOFSA, PISA, NISAP, NISD, vapor deposition of PDMS, and GraftFast have the potential to address these concerns by lowering synthetic cost and facilitating scaling up efforts. Scalability is a critical consideration, as industrial applications demand efficient and cost-effective manufacturing processes. Some techniques, like thermal vapor deposition and nonsolvent precipitation, appear well-suited for scaling up due to their simplicity and rapid deposition. Approaches involving custom macromolecular initiators may be less favorable for large-scale production due to the time and expense involved in each step, although these methods yield the most control over the final properties of the material. Another key challenge is ensuring the uniformity and stability of the polymer coatings are retained during scale-up, which are crucial for the consistent performance of PEFs. Efforts to scale up PEF production are still in their early stages, with limited attempts reported. As the demand for these hybrid materials in industrial applications grows, progress toward large-scale syntheses and experiments will become more important.

Exploring new polymer-framework combinations that can operate under extreme conditions, such as high temperatures and pressures, will broaden the application scope of PEFs. Another promising research direction is the integration of PEFs with other emerging technologies, such as sensing and catalysis, to develop multifunctional materials that can address multiple challenges in energy and environmental sectors simultaneously. Innovative fabrication techniques are needed to integrate PEFs into devices such as gas separation membranes, adsorbent beds, or sensors, ensuring the mechanical stability and longevity of these devices in real-world applications.

The long-term stability of PEFs under operational conditions, including exposure to varying temperatures, pressures, and gas compositions, is not well understood. Both the framework and the polymer components may undergo degradation or physical changes over time, which could compromise the material’s performance. Future research should focus on assessing the long-term stability of PEFs and developing strategies for their regeneration or recycling. This could include the study of self-healing polymers or easily replaceable polymer coatings to extend the lifetime of PEF-based systems. Research could also explore the use of biodegradable or biobased polymers for encapsulation, reducing the ecological footprint of PEFs. Additionally, life cycle assessments should be conducted to evaluate the overall environmental impact of PEF-based technologies, guiding the development of more sustainable solutions.

Overall, the field of PEFs is rapidly evolving, with a wide range of coating methods yielding well-characterized coatings with differing properties. The effects of polymer coatings on porosity and functionality vary depending on the coating mechanism, design, and intended application, underscoring the need for further characterization to expand fundamental understanding of PEFs. However, the remarkable capabilities of PEFs and the progress made in streamlining their synthesis in recent years hold great promise.
